# Evidence of ideal excitonic insulator in bulk MoS_2_ under pressure

**DOI:** 10.1073/pnas.2010110118

**Published:** 2021-03-23

**Authors:** S. Samaneh Ataei, Daniele Varsano, Elisa Molinari, Massimo Rontani

**Affiliations:** ^a^Consiglio Nazionale delle Ricerche - Istituto Nanoscienze, 41125 Modena, Italy;; ^b^Dipartimento di Scienze Fisiche, Informatiche e Matematiche, Università degli Studi di Modena e Reggio Emilia, 41125 Modena, Italy

**Keywords:** first-principles many-body perturbation theory, excitonic insulator, two-dimensional materials, ferroelectricity, Bose–Einstein condensation

## Abstract

Spontaneous condensation of excitons is a long-sought phenomenon analogous to the condensation of Cooper pairs in a superconductor. It is expected to occur in a semiconductor at thermodynamic equilibrium if the binding energy of the excitons—electron (e) and hole (h) pairs interacting by Coulomb force—overcomes the band gap, giving rise to a new phase: the “excitonic insulator” (EI). Transition metal dichalcogenides are excellent candidates for the EI realization because of reduced Coulomb screening, and indeed a structural phase transition was observed in few-layer systems. However, previous work could not disentangle to which extent the origin of the transition was in the formation of bound excitons or in the softening of a phonon. Here we focus on bulk ${MoS}_{2}$ and demonstrate theoretically that at high pressure it is prone to the condensation of genuine excitons of finite momentum, whereas the phonon dispersion remains regular. Starting from first-principles many-body perturbation theory, we also predict that the self-consistent electronic charge density of the EI sustains an out-of-plane permanent electric dipole moment with an antiferroelectric texture in the layer plane: At the onset of the EI phase, those optical phonons that share the exciton momentum provide a unique Raman fingerprint for the EI formation. Finally, we identify such fingerprint in a Raman feature that was previously observed experimentally, thus providing direct spectroscopic confirmation of an ideal excitonic insulator phase in bulk ${MoS}_{2}$ above 30 GPa.

## Significance

We claim that ${MoS}_{2}$ under pressure becomes the long-sought “excitonic insulator” (EI). This is a permanent condensate of excitons, electron–hole pairs bound by Coulomb interaction, which form in the absence of optical excitation. A surge of experimental claims has recently addressed layered materials, because of reduced Coulomb screening. However, the transition to the putative EI is ubiquitously accompanied by the softening of a phonon inducing a structural change; therefore it remains unclear whether the observed phase is excitonic or instead stabilized by electron–phonon interaction. Our calculations show that ${MoS}_{2}$ for a range of applied pressure is unstable against the generation of excitons but stable against lattice distortion: We predict that the EI is an antiferroelectric, electronic density wave.

The long-sought excitonic insulator (EI) is a permanent Bose–Einstein condensate of excitons in the absence of optical excitation, hosted in a narrow-gap semiconductor or a semimetal (1–4). As the exciton condensate shares similarities with the superconductor ground state (5), it may exhibit macroscopic quantum coherence and exotic low-energy excitations (6–12). These intriguing features are linked to the arbitrariness of the phase of the condensate wave function, φ (defined in Eq. **2** below): Whereas in the superconductor this phase degeneracy is protected by the conservation of electronic charge, in the EI it is contingent on the preservation of excitons (7, 11) and hence lifted by those terms in the Hamiltonian that annihilate or create e-h pairs. This is the case of e-phonon (13) and spin–orbit (14) interactions, which pin φ while hybridizing conduction and valence bands (remarkably, the combination of spin–orbit coupling and other factors may lead to a topological insulator whose character is inherited by the excitonic state) (14). Other mechanisms that act as sources/sinks for excitons include interband hybridization and Coulomb interaction terms allowed by symmetry (11), disorder (15), and environmental fluctuations of the electrostatic potential (8). So far, the most accomplished EIs were realized in bilayer heterostructures in the presence of a magnetic field, requiring both low temperature and complex engineering to maximize the impact of e-h correlations as well as the degeneracy of φ (10, 16). A related concept aims to achieve the temporary condensation of indirect excitons, made of spatially separated e and h, through the optical pumping of artificial bilayers designed to maximize the exciton lifetime (17–19).

Recently, layered materials (20–23) renewed the promise of the EI because of the enhanced Coulomb interactions, and hence exciton binding, due to their reduced dimensionality. In particular, the indirect character of excitons—in reciprocal (20, 21) and real (22, 23) space for ${TiSe}_{2}$ and ${Ta}_{2}{NiSe}_{5}$, respectively—led to inherently weaker screening and hence stronger e-h attraction, thus potentially stabilizing the EI phase. In those systems, the putative transition to the EI was accompanied by a lattice instability (24–28) when lowering the temperature—a singularity in the phonon density of states at vanishing energy—that in turn created e-h pairs through e-phonon interaction. In contrast, the transition to the ideal EI is purely electronic, with only small adjustments of the lattice (4, 29).

Here, we follow an early suggestion by Hromadová et al. (30) and focus on bulk ${MoS}_{2}$ under hydrostatic pressure (30–33). We use many-body perturbation theory from first principles (34, 35) to demonstrate that ${MoS}_{2}$ is unstable against exciton condensation but stable against lattice distortion. Building a self-consistent effective-mass model on top of ab initio calculations, we show that the true ground state is an ideal, antiferroelectric EI with a distinctive Raman fingerprint that has already been observed (36).

**Fig. 1.**
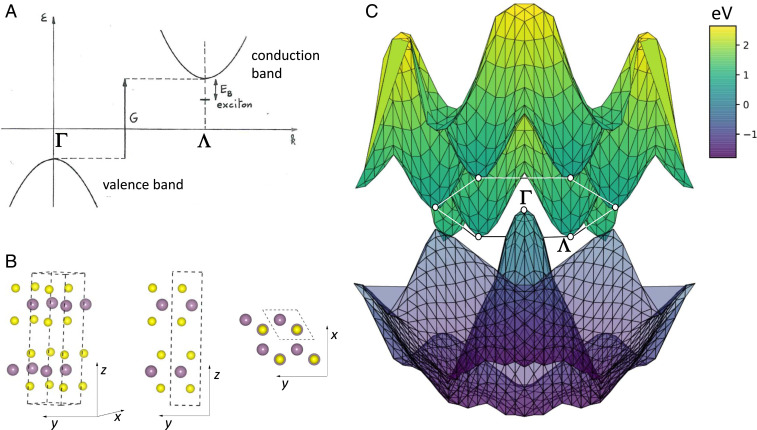
Indirect-gap ${MoS}_{2}$ as a candidate excitonic insulator. (*A*) Sketch of the excitonic insulator instability, adapted from Walter Kohn’s original proposal (4). An exciton binds an electron at the conduction band bottom, located at Λ in **k** space, with a hole at the valence band top at Γ. If the exciton binding energy, $E_{b}$, is larger than the indirect gap, G, then the system is unstable against the spontaneous generation of excitons. The reconstructed many-body ground state—a condensate of excitons at thermodynamic equilibrium—is the excitonic insulator. (*B*) Model of the 2$H_{c}$ crystal structure from different views. The violet (yellow) color labels Mo (S) atoms. The dashed frame appearing in side and top views is the unitary cell of the layered structure, with a and c being the in- and out-of-plane lattice constants, respectively. (*C*) Lowest conduction and topmost valence energy band as a function of wave vector in the $k_{z}=0$ plane, as obtained from first-principles many-body perturbation theory (GW) at a pressure of 34 GPa.

In bulk ${MoS}_{2}$, the pressure (P) closes the indirect gap, G, between the top of the filled valence band, located at the center of the Brillouin zone (Γ point), and the bottom of the six-degenerate valleys of the empty conduction band—placed at Λ points (approximately midway between Γ and K; see Fig. 1*C* for P= 34 GPa). The energy landscape along one of the ΓΛ cuts (sketched in Fig. 1*A*) favors the Coulomb binding of an e, located at Λ, with an h, placed at Γ, creating an exciton of finite momentum |**q**| =ΓΛ and binding energy $E_{b}$. Whereas ordinarily $E_{b}<G$, it may occur that $E_{b}>G$ above a critical pressure, a condition that makes the semiconductor unstable against the condensation of excitons. This is actually the case, as we show below from first principles.

So far, ultrahigh pressure has been used as a handle to make MoS_2_ superconducting (33) (at P∼ 90 GPa), although the pairing mechanism remains unclear (37–39). The putative EI must be searched at lower pressure (P∼ 25 GPa), close to the semiconductor–semimetal transition that was observed by several groups (31, 32, 40–42). Near this boundary, theory (30, 43)—including our own calculations (*SI Appendix*, Fig. S1)—predicts an isostructural transition from the 2$H_{c}$ (Fig. 1*B*) to the 2$H_{a}$ (*SI Appendix*, Fig. S2) phase, which does not affect the crystal space group $D_{6h}^{4}$, as the two structures transform into each other through the sliding of the layers in the unit cell (the layer unit is made of one Mo and two S atoms, represented respectively by violet and yellow balls in the sketch of Fig. 1*B*). Raman and X-ray spectroscopic observations (31, 33, 36, 41, 44) suggest that 2$H_{c}$ and 2$H_{a}$ phases coexist in diamond-anvil cells, in a range that varies between 25 and 50 GPa in powders but has narrower extension (∼4 GPa) in single crystals. Importantly, we find that both 2$H_{c}$ and 2$H_{a}$ polytypes experience a similar excitonic instability—unrelated to the structural transition, as the electronic bands of the two phases are basically identical close to the Fermi energy. Below, we discuss the 2$H_{c}$ stacking and leave the analysis of 2$H_{a}$ to *SI Appendix*, Figs. S3 and S4.

## Results

**Fig. 2.**
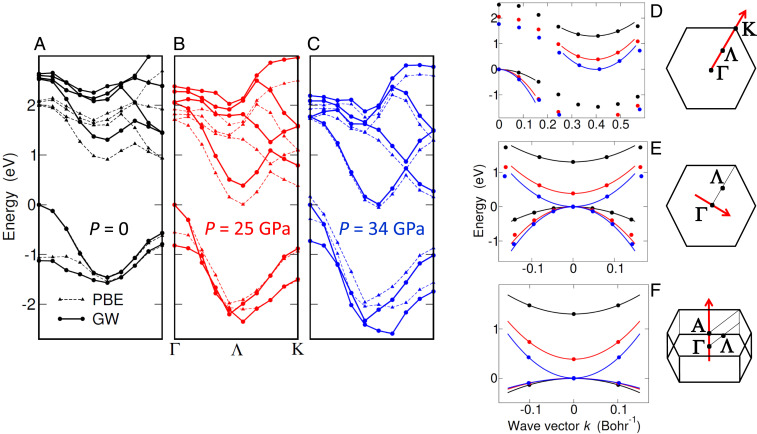
Closing the gap by applying pressure. (*A–C*) Band structure along the Γ−Λ−K cut of the Brillouin zone at pressure P= 0 (*A*), 25 (*B*), and 34 GPa (*C*). Band energies obtained from first principles including the quasiparticle GW corrections beyond DFT (circles) are compared to bare DFT data (triangles, PBE functional). Lines are guides to the eye. (*D*–*F*) Band dispersion of conduction and valence bands close to Λ and Γ points, respectively, for P= 0 GPa (black color), 25 GPa (red), and 34 GPa (blue). In *E* and *F* the conduction band has been rigidly translated by the wave vector $-\vec{\Gamma \Lambda }$. GW predictions (dots) are shown together with effective-mass fits (curves). The directions of the cuts (shown as red arrows in the Brillouin zone) are the principal axes of the effective-mass tensor, two being in the $k_{z}=0$ plane (*D* and *E*) and one being parallel to the $k_{z}$ axis (*F*).

The indirect gap of 2$H_{c}$–${MoS}_{2}$ is sensitive to pressure, as its value drops from 1.31 eV at P=0 (Fig. 2*A*) to only 9 meV at P=34 GPa (Fig. 2*C*), close to the semimetal limit. With respect to the accurate band structure calculated within the GW approximation (circles in Fig. 2 *A*–*C*) (*Materials and Methods*), density functional theory (triangles) underestimates the gap of about 0.4 eV at P=0. However, as pressure reduces the out-of-plane lattice parameter c (*SI Appendix*, Fig. S1), forcing sulfur orbitals belonging to adjacent layers to overlap (45), virtual e-h pairs start tunneling among layers, screening effectively Coulomb interaction at long wavelength. This reduces the GW energy correction to the density functional theory (DFT) bandgap, as evident in Fig. 2*C*. Consistently, the conduction band increases its dispersion along the $k_{z}$ direction (Fig. 2*F*), as well as the other axes of the effective mass tensor (Fig. 2 *D* and *E*; dots and lines are GW data and effective-mass fits, respectively). Overall, the semiconductor becomes progressively more isotropic as it turns into a semimetal, losing its two-dimensional character.

### Exciton Binding and Instability.

**Fig. 3.**
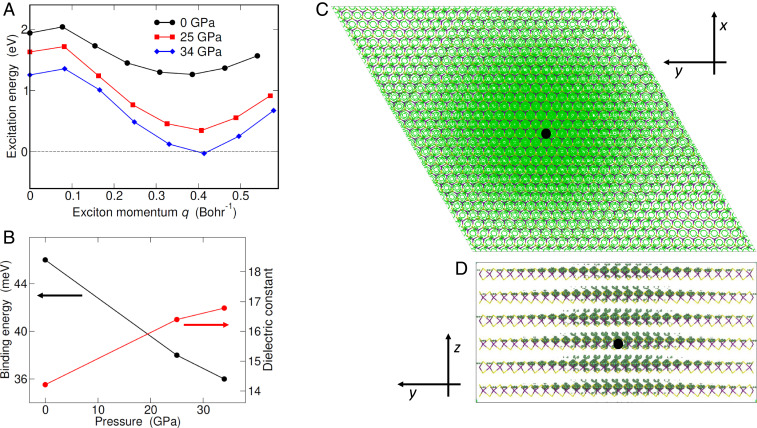
Excitonic instability. (*A*) Excitation energy of the lowest exciton vs. center-of-mass momentum **q** along the ΓK direction. Data are obtained from first principles for P= 0 (dots), 25 (squares), and 34 GPa (diamonds). Note that the K point position expressed in units of ${Bohr}^{-1}$ shifts with P. Solid lines are guides to the eye. At P= 34 GPa the excitation energy is negative for q=ΓΛ, which points to the instability against exciton condensation (the dashed line highlights the energy zero). (*B*) Binding energy of the exciton having momentum q=ΓΛ (black circles, left vertical axis) and macroscopic static dielectric constant (red circles, right axis) vs. P. The latter is obtained through the inverse dielectric matrix, as $1/\;{\left[\epsilon^{-1}\left(q=0\right)\right]}_{G=G^{\prime }=0}$ (**G** is the reciprocal lattice vector). (*C* and *D*) Wave function square modulus of the lowest exciton with q=ΓΛ at P=0. The plot shows the conditional probability to locate the bound electron (green contour map), provided the hole position is fixed (black dot), either in (*C*) or out (*D*) of plane. The violet (yellow) color in the stick-and-ball skeleton points to Mo (S) atoms.

The exciton candidate for the instability has a finite center-of-mass momentum **q**; i.e., it travels in space. We compute its excitation energy—the difference between the GW bandgap and the binding energy—by solving the Bethe–Salpeter equation from first principles (*Materials and Methods*). The dispersion exhibits a dip for q=Λ, whose energy is first positive at P=0 (1.26 eV, black dots in Fig. 3*A*) but then quickly lowers with P, eventually changing sign close to the semimetal threshold (−27 meV at P=34 GPa, blue diamonds). This negative value signals that excitons spontaneously form, which leads to a reconstructed many-body phase of lower energy.

The softening of the exciton shown in Fig. 3*A* validates from first principles the seminal prediction by des Cloizeaux (2): The binding energy remains finite even if the gap vanishes, as explicitly shown in Fig. 3*B* (black dots). The reason is that conduction and valence band profiles are almost unaffected by P (Fig. 2), as the band edges are displaced in **k** space, which prevents the macroscopic dielectric constant from diverging (red dots in Fig. 3*B*). Were the closing gap direct, metal-like screening would dissociate the exciton.

The square modulus of the exciton wave function is illustrated in Fig. 3 *C* and *D*, as the conditional probability density to locate the bound electron (green contour map), provided the hole is fixed (black dot). Note that the center-of-mass motion does not appear in this frame. The probability extends tens of angstroms—the feature of Wannier excitons familiar from bulk semiconductors—both in and out of plane, as apparent in Fig. 3 *C* and *D*, respectively (the Bohr radius is 50 Å at 34 GPa, as shown in *SI Appendix*, Fig. S5). The exciton becomes lighter and more isotropic with pressure, i.e., more delocalized in real space (here shown at P=0).

### Two-Band Model.

The major source of numerical error is the finite sampling of the Brillouin zone (14), since the exciton is significantly localized in **k** space while the computational load prevents us from refining the mesh (*Materials and Methods*). However, the specific features of the exciton provide us with a workaround, since 1) the wave function is spanned essentially by those e and h states that are close to the edges of the lowest conduction and highest valence band, respectively (Fig. 1*A*) and 2) the spin degree of freedom is irrelevant, the exciton energy being fourfold degenerate within numerical accuracy (spin–orbit coupling is fully included in the calculation). Therefore, we may afford ultradense **k**-space sampling by replacing the first-principles Bethe–Salpeter equation with its spinless two-band counterpart within the effective mass approximation (35), the mass tensor being extracted from Fig. 2 *D*–*F* and the dielectric constant from Fig. 3*B* (*Materials and Methods* and *SI Appendix*, Fig. S5). The resulting excitation energy, at the semimetal threshold, is ≈−8 meV.

### The Excitonic Insulator Phase.

Close to the semiconductor–semimetal boundary, the ground state undergoes a reconstruction from the “normal” phase, $\left|\Phi_{0}\right\rangle$, which is either insulating or semimetallic, to the excitonic insulator, $\left|\Psi_{\text{EI}}\right\rangle$. In the following, we highlight the essential features of $\left|\Psi_{\text{EI}}\right\rangle$ within the simpler two-band model (as a mnemonic, we adopt the apex “0” to identify quantities of interest defined within this model). Then, we take into account the EI multivalley nature by adapting the theory first proposed for the candidate material ${TiSe}_{2}$ (46).

Within the two-band model (3), $\left|\Psi_{\text{EI}}^{0}\right\rangle$ is formally analogous to the superconductor wave function (5),

$$\left|\Psi_{\text{EI}}^{0}\right\rangle =\underset{k}{\prod }\left[u_{k}^{0}+v_{k}^{0}e^{-i\varphi }\;{\hat{b}}_{k}^{+}{â}_{k}\right]\left|\Phi_{0}\right\rangle \;,$$ [1]

provided the Cooper pairs of the metal are replaced with the e-h pair excitations of the normal state, ${\hat{b}}_{k}^{+}{â}_{k}\left|\Phi_{0}\right\rangle$. Here ${\hat{b}}_{k}^{+}$ creates an electron with momentum **k** $+\;\vec{\Gamma \Lambda }$ and energy $\varepsilon_{b}\left(k\right)$ in the conduction band, ${â}_{k}$ annihilates an electron with momentum **k** and energy $\varepsilon_{a}\left(k\right)$ in the valence band, $u_{k}^{0}$ and $v_{k}^{0}$ are positive coherence factors [${\left(u_{k}^{0}\right)}^{2}+{\left(v_{k}^{0}\right)}^{2}=1$], and φ is the phase of the condensate wave function, $\zeta_{k}^{0}=u_{k}^{0}v_{k}^{0}e^{i\varphi }=\Delta_{k}^{0}/2E_{k}$, with $\Delta_{k}^{0}$ being the excitonic gap function and $E_{k}={\left\{{\left[\varepsilon_{b}\left(k\right)-\varepsilon_{a}\left(k\right)\right]}^{2}/4+|\Delta_{k}^{0}{|}^{2}\right\}}^{1/2}$. The value of φ is—ideally—arbitrary and solely fixed by the spontaneous breaking of the conservation law for e-h pairs, as

$$\left\langle \Psi_{\text{EI}}^{0}\right|{\hat{b}}_{k}^{+}{â}_{k}\left|\Psi_{\text{EI}}^{0}\right\rangle =\left|\zeta_{k}^{0}\right|e^{i\varphi }.$$ [2]

**Fig. 4.**
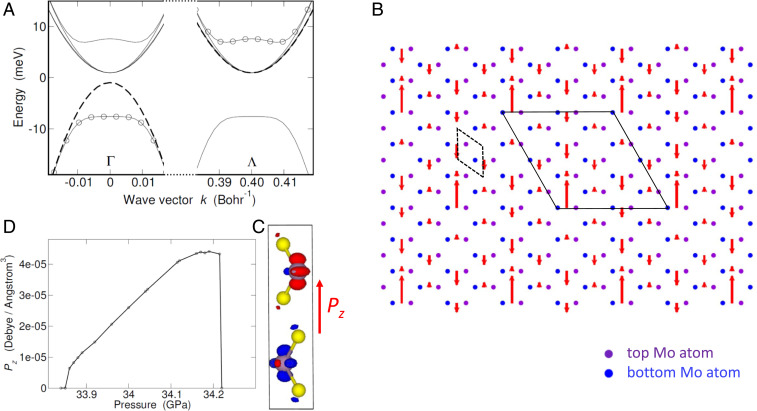
Antiferroelectric excitonic insulator. (*A*) Band structure of the excitonic (solid curves) and pristine (dashed curves) insulator along one of the six equivalent Γ−Λ directions in the $k_{z}=0$ plane of the Brillouin zone at P= 34 GPa. The original conduction bands are folded from Λ valleys to Γ and renormalized together with the valence band. The new band structure at Γ is replicated at Λ, since both Γ and Λ points belong to the EI reciprocal lattice. Apart from spin degeneracy, EI renormalized bands exhibit an additional orbital degeneracy reminiscent of the pristine multivalley structure: Bands (solid curves) from top to bottom are respectively one-, three-, two-, and onefold degenerate, respectively. The twofold degenerate band, which overlaps with the pristine conduction band (dashed curve), is actually split due to the tiny anisotropy of $\Delta_{k}$ in the $k_{x}$, $k_{y}$ plane (splitting hardly visible in the plot). Circles point to EI conduction and valence bands obtained within the two-band model. (*B*) Antiferroelectric structure. A permanent out-of-plane electric dipole, $P_{z}\left(x,y\right)$, spontaneously develops and exhibits an in-plane modulation that breaks inversion symmetry. This dipole, which averages to zero over the unitary cell of the superstructure (solid frame), is perpendicular to the plane and depicted as a red arrow of varying sign and modulus. The superstructure cell contains 72 atoms against 6 of the original cell (dashed frame) (here ΓΛ≈2π/(3a)). Violet and blue dots are Mo atoms, respectively, in the top and bottom layer (S atoms are not shown). (*C*) Overlap charge density of the periodic part of pristine conduction and valence band Bloch states, respectively, at Λ and Γ, shown in the 2$H_{c}$ cell. The red (blue) color points to a surplus (deficit) of charge. The depicted charge displacement, which is associated with the polarization of condensed excitons, is the origin of the permanent dipole $P_{z}$ shown in *B*. (*D*) Maximum local value of $P_{z}$ vs. P.

The EI band structure is obtained by solving the pseudo-Bethe–Salpeter equation for $\zeta_{k}^{0}$ self-consistently,

$$2E_{k}\;\zeta_{k}^{0}-\underset{k^{\prime }}{\sum }W\left(k-k^{\prime }\right)\;\zeta_{k^{\prime }}^{0}\;=\;0,$$ [3]

where W(q) is the screened Coulomb interaction and the minimum value of $2E_{k}$ is the bandgap (*Materials and Methods*). Reassuringly, Eq. **3** turns into the Bethe–Salpeter equation for the zero-energy exciton at the onset of the EI phase ($\Delta_{k}^{0}\to 0+$). As a consequence of the condensation energy gain, the EI conduction and valence bands (circles in Fig. 4*A*) are flattened and distorted with respect to those of the pristine semiconductor (dashed curves), the gap widening by ≈15 meV at P=34 GPa.

### Multivalley Effects.

As e-h pairs may be formed by exciting an electron from the valence band to any one of the six conduction band valleys, $\Lambda_{i}$, the condensate wave function is multicomponent (46), $\left\langle \Psi_{\text{EI}}\right|{\hat{b}}_{ik}^{+}{â}_{k}\left|\Psi_{\text{EI}}\right\rangle =\zeta_{ik}$, with ${\hat{b}}_{ik}^{+}$ creating an electron with momentum **k** $+\;{\vec{\Gamma \Lambda }}_{i}$ and energy $\varepsilon_{ib}\left(k\right)$ in the ith valley (i=1,…,6). In principle, one must solve up to six coupled equations for $\zeta_{ik}$ to account for the distortion of the condensate in **k** space, due to intervalley coupling. Nevertheless, we note that $\Delta_{k}^{0}$ has hardly any angular dependence in the $k_{x}$, $k_{y}$ plane (the maximum amplitude of the azimuthal modulation is smaller than 0.07 meV) (*SI Appendix*, Fig. S6), whereas $\varepsilon_{ib}\left(k\right)$ depends on the angle between ${\vec{\Gamma \Lambda }}_{i}$ and ($k_{x},k_{y}$) due to mass anisotropy. As Coulomb interaction protects the cylindrical symmetry of $\zeta_{ik}$, and since the bare-band anisotropy has negligible effect at valley bottom k≈0 (where the value of $\zeta_{ik}$ is largest), we neglect the azimuthal dependence of $\zeta_{ik}$ and obtain (*Materials and Methods*)

$$\zeta_{ik}=\left\langle \Psi_{\text{EI}}\right|{\hat{b}}_{ik}^{+}{â}_{k}\left|\Psi_{\text{EI}}\right\rangle =\frac{1}{\sqrt{6}}\;u_{k}^{0}v_{k}^{0}\;e^{i\varphi_{i}},\;i=1,\ldots ,6.$$ [4]

Here only the magnitude of $\zeta_{ik}$ is fixed (from the self-consistent solution of Eq. **3**), whereas the six phases $\varphi_{i}$ remain undetermined. This is sufficient to compute the band structure of the EI (Fig. 4*A*), as the ground-state energy is independent from $\varphi_{i}$ (*Materials and Methods*).

There are now one valence and six conduction bands (solid thin lines in Fig. 4*A*), in place of the two bands (circles) of the superconductor-like model. Some of the conduction bands are degenerate, the degeneracy being respectively one, three, two, and one, from the topmost conduction to the valence band. Importantly, the band structure at Γ is replicated at Λ, as the electronic charge exhibits a supermodulation in real space that we discuss below, the corresponding unit cell (solid frame in Fig. 4*B*) being larger than the cell of the crystal lattice (dashed frame). As a consequence, bands are folded into the smaller Brillouin zone (*SI Appendix*, Fig. S6), changing the gap from indirect to direct. Only the valence and topmost conduction bands repel each other, in agreement with the two-band model (circles), whereas the remaining bands, which are unaffected by the presence of the exciton condensate, replicate at Γ the bare valleys and hence reduce the direct gap. Since the location of the valence band top is slightly displaced from Γ along the $k_{z}$ axis (*SI Appendix*, Fig. S7), by ∼0.02$\;{Bohr}^{-1}$, the actual EI gap is indirect and around ∼ 5 meV, smaller than the direct gap at Γ. Note that in Fig. 4*A* the twofold degenerate band, which almost overlaps with the bare conduction band (dashed curve), splits due to the tiny anisotropy of $\Delta_{k}$ in the $k_{x}$, $k_{y}$ plane (the splitting is hardly visible in the plot).

### Antiferroelectric Excitonic Insulator.

The EI ground state is invariant under time reversal, and hence the phases of the condensate components that live in two antipodal valleys must have opposite sign (modulus a multiple integer of 2π); i.e., $\varphi_{1}=-\varphi_{4}$, $\varphi_{3}=-\varphi_{6}$, and $\varphi_{5}=-\varphi_{2}$ (*SI Appendix*, Fig. S6 and *Materials and Methods*). This constraint leads to the formation of a purely electronic, self-sustained charge density wave, Δϱ(r), which breaks the inversion symmetry of the pristine crystal (the proof is given in *Materials and Methods*). The total wave Δϱ is the coherent superposition of three contributions, $\Delta \varrho =\Delta \varrho_{1,4}+\Delta \varrho_{3,6}+\Delta \varrho_{5,2}$, each one originating from a couple of antipodal valleys. For example,

$$\begin{array}{ccc} \Delta \varrho_{1,4}\left(r\right) & = & \frac{8}{\sqrt{6}}\left[\underset{k}{\sum }\;u_{k}^{0}v_{k}^{0}\right]\;\times \\ & & \text{Re}\;\left\{\psi_{\Gamma }\left(r\right)\;\psi_{\Lambda_{1}}^{*}\;\left(r\right)\;\exp\;\left[-i\left({\vec{\Gamma \Lambda }}_{1}\;\cdot \;r-\varphi_{1}\right)\right]\right\} \end{array}$$ [5]

exhibits the new periodicity $2\pi /|{\vec{\Gamma \Lambda }}_{1}|$ given by the momentum of those excitons that condense in valleys 1 and 4, and similarly $\Delta \varrho_{3,6}$ and $\Delta \varrho_{5,2}$ display an analogous modulation along directions ${\vec{\Gamma \Lambda }}_{3}$ and ${\vec{\Gamma \Lambda }}_{5}$ with phase shifts $\varphi_{3}$ and $\varphi_{5}$, respectively. Here $\psi_{\Gamma }$ and $\psi_{\Lambda_{1}}$ are the periodic envelopes of Bloch states respectively at Γ and $\Lambda_{1}$, $\psi_{\Lambda_{4}}=\psi_{\Lambda_{1}}^{*}$, and the spin has been factored out, since the lattice space group contains a center of inversion and a unique z axis (47). It is clear that the total amount of charge displaced from the pristine background, as well as the amplitude of the charge modulation, is driven by the condensate through $\sum_{k}\;u_{k}^{0}v_{k}^{0}$.

Importantly, the arbitrariness of the phases $\varphi_{1}$, $\varphi_{3}$, and $\varphi_{5}$ points to a huge, continuous degeneracy of the ground state. Since the effect of any given two arbitrary phases is merely to rigidly shift the charge pattern Δϱ with respect to the frame origin (*Materials and Methods*), in the following we take $\varphi_{1}=\varphi_{3}=\varphi_{5}=0$. The resulting density wave is slightly distorted in the generic case, in which all three phases take arbitrary values (discussion below).

Fig. 4*C* shows the overlap charge density of the envelopes obtained from first principles, $\sum_{\sigma }\psi_{\Gamma \sigma }^{*}\left(r\right)\;\psi_{\Lambda_{1}\sigma }\left(r\right)\;+\;\text{c.c.}$, which is proportional to $\Delta \varrho_{1,4}$ in the unit cell at the origin (we have added the subscript σ to ψ since the numerical envelopes are generically spinors in the presence of spin–orbit coupling). The density wave shows an asymmetric pattern—transferring charge mainly between the two Mo atoms, which breaks the inversion symmetry with respect to the origin of the cell (the red [blue] contour map points to a surplus [deficit] of charge). This charge transfer sets a local electric dipole with an in-plane texture, $P_{1,4}\left(x,y\right)$, as $\Delta \varrho_{1,4}$ is modulated by $\exp\;\left[i\left({\vec{\Gamma \Lambda }}_{1}\;\cdot \;r\right)\right]$. This dipole may be regarded as the polarization of the excitons coherently built in the condensate (8). Since the contributions to the dipole due to the remaining valleys, $P_{3,6}$ and $P_{5,2}$, are obtained by rotating $P_{1,4}$ by respectively 2π/3 and −2π/3 along the z axis, the total dipole $P=P_{1,4}+P_{3,6}+P_{5,2}$ is parallel to the z axis. We evaluate this parallel component, $P_{z}$, through direct integration over the unit cell (Fig. 4*D* and *Materials and Methods*).

The overall charge pattern, $P_{z}\left(x,y\right)$, exhibits an antiferroelectric texture that breaks inversion symmetry. This is shown in Fig. 4*B*, where local dipoles, which point out of the plane, are depicted as red arrows having length proportional to $\left|P_{z}\right|$. The electric dipole averages to zero over the unitary cell of the superstructure (solid frame), which contains 72 atoms (with ΓΛ≈2π/(3a)) against 6 of the original cell (dashed frame). The reconstructed Brillouin zone, which is again hexagonal in the plane but rotated by π/6 (*SI Appendix*, Fig. S6*D*), is spanned by any two independent vectors chosen among the ${\vec{\Gamma \Lambda }}_{i}$s. In the generic, degenerate case that $\varphi_{1}$, $\varphi_{3}$, and $\varphi_{5}$ take arbitrary values, we expect a reduction of the maximum local value of $\left|P_{z}\right|$ up to 2/3, together with a variable tilt of the dipole in the plane.

### Semiconductor–Semimetal Cross-Over.

**Fig. 5.**
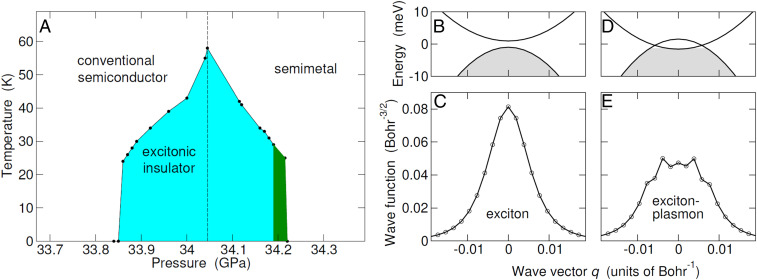
Excitonic insulator phase diagram. (*A*) Phase diagram in the P–T space. Lines are guides to the eye. The shadowed area highlighted in cyan (green) color is the excitonic gapped (gapless) phase. The vertical dashed line points to the semiconductor–semimetal boundary in the absence of excitonic effects. (*B*–*E*) Bare energy bands and wave function of the exciton driving the instability in the e-h center-of-mass frame, evaluated in reciprocal space along the ΓΛ direction. The e-h pair of wave vector q is made of a hole with momentum −q and an electron with momentum q+ΓΛ (in *B* and *D* the bare conduction band has been displaced by the vector $-\vec{\Gamma \Lambda }$ and the shadowed region highlights occupied states). Going from P=34 GPa (*B* and *C*) to P=34.12 GPa (*D* and *E*), a Fermi surface forms as conduction and valence band overlap in energy. Consequently, plasmonic features appear in the exciton wave function, the spectral weight accumulating close to the Fermi surface (*E*). In *D* the Fermi energy is negative as a consequence of the sixfold valley degeneracy at Λ.

The formation of a Fermi surface, made of six e pockets in the Λ valleys and one h pocket at Γ, signals the transition from the semiconductor (Fig. 5*B*) to the semimetal (Fig. 5*D*) occurring in the absence of excitonic effects. Fig. 5 *B* and *D* shows one of the conduction valleys, displaced by $-\vec{\Gamma \Lambda }$ in **k** space, and the valence band, the filled states being shadowed by gray color. As the free carriers populating the Fermi pockets effectively screen the e-h attraction, we replace the long-range Coulomb force W in Eq. **3** with the vertex interaction proposed by Kozlov and Maksimov (48) to establish self-consistently the range of the force; besides, we extrapolate P-dependent masses from first principles (*Materials and Methods*).

The resulting EI phase extends over a narrow interval of ≈0.35 GPa, reaching a maximum critical temperature of T≈60 K at P≈34.05 GPa, which is the semiconductor–semimetal boundary in the normal state (vertical dashed line in Fig. 5*A*). Importantly, the downward shift of the valence band shown in Fig. 4*A* opens/widens the gap over a pressure range that extends to values that would lead to a semimetal for $\left|\zeta_{ik}\right|=0$. In the P−T diagram of Fig. 5*A*, the gapped excitonic phase, highlighted as a shadowed cyan area, is the overwhelming part of the larger region that sustains a finite condensate of excitons, $\left|\zeta_{ik}\right|>0$. The remaining excitonic region—the thin green slice located between P∼ 34.19 and 43.22 GPa—is gapless (*SI Appendix*, Fig. S7) and ends on the semimetal frontier where $\left|\zeta_{ik}\right|=0$. Here the critical pressure is equivalent to an amount of free carriers (the density per species is $1.1\cdot 10^{-7}\;{Bohr}^{-3}$) comparable to the maximum number of excitons in the condensate ($2.2\cdot 10^{-7}\;{Bohr}^{-3}$). This overall behavior is in stark contrast with that of the EI candidate ${TiSe}_{2}$, which has a multivalley structure similar to that of ${MoS}_{2}$ but remains a semimetal due to the unintentional doping of Ti atoms (46).

**Fig. 6.**
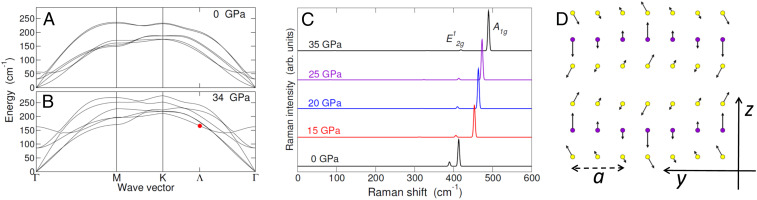
Phonon dispersion and Raman fingerprint. (*A* and *B*) Dispersion of the lowest-energy phonon modes for P=0 (*A*) and 34 GPa (*B*), respectively, computed from first principles. All modes harden with P. The red dot points to the lowest optical mode that is folded from Λ into Γ through the excitonic insulator phase transition. (*C*) Raman spectrum of the normal phase from first principles, for pressures P= 0, 15, 20, 25, and 35 GPa, respectively, from bottom to top. The peaks are broadened using Gaussians with a standard deviation of 2 ${cm}^{-1}$. The bright peak at lower (higher) frequency has $E_{2g}^{1}$ ($A_{1g}$) symmetry. The plot compares with figure 4b of ref. 36. (*D*) Displacement vectors for the mode labeled as a red dot in *B*, as viewed in the excitonic insulator reconstructed cell along the ΓΛ direction (parallel to the y axis in the adopted frame) (47). The superlattice constant is 3a. The violet (yellow) color labels Mo (S) atoms. This mode is Raman active and degenerate with the one folded from $\Lambda^{\prime }$ to Γ.

The exciton responsible for the instability of the conventional semiconductor exhibits a mixed transverse–longitudinal polarization (49), due to the small $C_{2}$ symmetry of the ΓΛ line (this is also the case of the displacement vectors of the vibrational mode of Fig. 6*D*). As one moves from the semiconductor to the semimetal, the exciton smoothly turns into a plasmon (4), as illustrated by the wave function in the e-h center-of-mass frame (*Materials and Methods*). Whereas in the semiconductor (Fig. 5*C*) the amplitude is Lorentzian-like in **k** space, similar to that of a familiar Wannier exciton in the bulk, in the semimetal it acquires plasmonic features, as the wave function accumulates close to the Fermi surface (Fig. 5*E*). Outside the EI phase, this exciton–plasmon dissolves into the continuum of e-h excitations. Note that there may be other long-lived interband plasmons, since small gaps open in the e-h energy continuum due to the degeneracy of Λ valleys. Were there only one valley, then the Fermi energy would be at the crossing of a and b bands (ignoring the mass anisotropy; cf. Fig. 5*D*) and the e-h excitation spectrum would be gapless.

### Raman Fingerprint.

Were ion displacements responsible for the building of electric dipoles in place of excitons, the frequency of the phonon of momentum **q** $=\vec{\Gamma \Lambda }$ and consistent symmetry would soften (or at least exhibit a dip) at the onset of the new phase (50). The phonon dispersion obtained from first principles, respectively at P=0 (Fig. 6*A*) and 34 GPa (Fig. 6*B*), shows the opposite behavior, with all low-energy modes hardening with P (see *Materials and Methods* and *SI Appendix*, Fig. S8 for the 2$H_{a}$ phase). Therefore, the antiferroelectricity has a purely electronic origin. This prediction is consistent with recent diffraction measurements, which ruled out any periodic lattice distortion above 40 K (44).

The evolution of Raman spectrum with pressure, as obtained from first principles in Fig. 6*C* (structure 2$H_{c}$) and *SI Appendix*, Fig. S9 (2$H_{a}$), compares with observed data with the exception of the $E^{\prime }$ peak at 174 ${cm}^{-1}$ (figure 4b in ref. 36), which appears below 150 K and above 30 GPa but is missed by the theory for the normal phase. Cao et al. (36) proposed this mode is a transverse acoustic phonon of finite momentum, which becomes bright at the onset of a charge density wave, due to the reconstruction of the Brillouin zone. Whereas the first-principles spectrum for the excitonic phase is presently out of reach, below we confirm the essence of Cao’s explanation by identifying $E^{\prime }$ as the lowest optical phonon at Λ. This is the fingerprint of the antiferroelectric charge density wave associated with exciton condensation.

The symmetry group of the antiferroelectric ground state depicted in Fig. 4*B* includes only the identity operation. Therefore, all 216 vibrational modes are in principle infrared and/or Raman active. However, since the EI critical temperature is relatively low and the $E^{\prime }$ peak is extremely bright, we expect that the new mode is an optical phonon of momentum Λ, which is Raman active through the folding into the zone center and strongly couples with P. Since P(x,y) originates everywhere in the cell from the interlayer vertical displacement of the charge between two neighbor Mo atoms, it will mainly couple with those optical oscillations of Mo atoms that occur along the z axis. In fact, these vibrations linearly change the Mo-Mo distance and hence the local dipole strength, whereas the amount of displaced charge, which is ruled by the long-range part of Coulomb interaction, changes weakly with the oscillation. From direct inspection of phonon eigenvectors, there is one candidate only below 400 ${cm}^{-1}$, i.e., the lowest optical mode of frequency 164 ${cm}^{-1}$ located at Λ, which is highlighted by a red dot in Fig. 6*B*. As shown by the displacement vectors in the EI reconstructed cell displayed in Fig. 6*D*, the Mo atoms oscillate out of phase along the z direction with an in-plane modulation of period 3a along the $\vec{\Gamma \Lambda }$ direction (parallel to the vertical axis of Fig. 4*B*), hence matching the periodicity of $P_{z}\left(x,y\right)$ in the plane. This superlattice vibration is twice degenerate, due to the additional folding of the phonon with independent wave vector ${\vec{\Gamma \Lambda }}^{\prime }$. Note that the observed intensity of the $E^{\prime }$ mode is constant up to 60 K, which compares with the EI critical temperature. In summary, the $E^{\prime }$ mode points to the excitonic insulator in the P−T space.

## Discussion

Both $2H_{c}$ and $2H_{a}$ phases coexist (31–33, 41) in the region of visibility of the $E^{\prime }$ mode, which extends between 30 and 50 GPa at 5 K (36). The lower bound agrees with our prediction, since in the $2H_{a}$ structure the EI sets in at P∼28 GPa (*SI Appendix*, Fig. S4) with a mode frequency of 166 ${cm}^{-1}$ (*SI Appendix*, Fig. S8). The upper bound of 50 GPa is larger than our expectation of ∼34 GPa for the $2H_{c}$ phase. However, recent diffraction measurements on single crystals (44), although available only at temperatures higher than 40 K, suggest that the critical upper pressure could be actually much lower, being artificially enhanced in powders due to the deviatoric stress field applied to randomly oriented crystallites.

In addition, other Raman features unexplained so far (36) point to the EI scenario: 1) the observation of modes supposedly forbidden or silent and 2) the anomalous frequency variation of the out-of-plane $A_{1g}$ mode accompanying the onset of the $E^{\prime }$ mode. Since the understanding of the available electrical transport measurements (31, 36) is complicated by the mixture of phases in the high-pressure cell, we do not speculate on the origin of the resistivity peak that was tentatively assigned (31) to the EI.

The huge degeneracy of the EI ground state, associated with condensate phases $\varphi_{1}$, $\varphi_{3}$, and $\varphi_{5}$, points to the emergence of acoustic-like electronic excitations—collective phase modes that, if gapless, would manifest exciton superfluidity (4). Within the two-band model of an isotropic semimetal, Kozlov and Maksimov (51) predicted that the “excitonic sound” velocity, $c_{\text{exciton}}=\hbar k_{F}{\left(3m_{a}m_{b}\right)}^{-1/2}$, is proportional to the Fermi wave vector in the normal phase, $k_{F}$ ($m_{a}$ and $m_{b}$ are valence and conduction band masses). By taking average values at the EI/semimetal boundary, we estimate $c_{\text{exciton}}∼2\cdot 10^{4}$ m/s, which is much higher than the sound velocity of the stiffest acoustic phonon branch (Fig. 6*B*), $c_{\text{phonon}}∼8\cdot 10^{3}$ m/s. Therefore, the phase mode of the exciton condensate should be experimentally accessible, even if it might be gapped due to the mechanisms mentioned in the Introduction and not included in our analysis of the EI phase.

## Conclusion

In summary, we have demonstrated that a real excitonic insulator phase sets in between the semiconducting and semimetallic phases of ${MoS}_{2}$, building on calculations from first principles and available spectroscopic data. These findings call for further investigation of some fascinating possibilities. A first question is the manifestation of the macroscopic quantum coherence of the exciton condensate, which might occur through the observation of low-lying collective modes associated with the oscillation of the condensate phase φ(r,t). Another issue is whether the superconductivity observed above 90 GPa is related to the excitonic phase, as the overscreening action of surviving exciton–plasmons might act as unconventional glue for Cooper pairs. We hope our study may stimulate further work along these paths.

## Materials and Methods

### Computational Details of Ground-State Calculation from First Principles.

The lattice parameters and the ground-state electronic structure for the three values of pressure were obtained within DFT, with a plane wave basis set as implemented in the Quantum ESPRESSO package (52, 53), using the generalized gradient approximation Perdew–Burke–Ernzerhof (PBE) parameterization (54). A kinetic energy cutoff of 100 Ry was adopted for the wave functions, and fully relativistic norm-conserving pseudopotentials (55) were used to take into account spin–orbit interaction. van der Waals interactions, included by using the Grimme approximation method, were found to be relevant only at zero pressure, as already shown in ref. 30.

### Phonons.

Phonon dispersions were calculated by using a density functional perturbation theory approach (56). We used a 10 × 10 × 3 Monkhorst–Pack grid for the integration in the Brillouin zone; the dynamical matrix at a given point of the Brillouin zone was obtained from a Fourier interpolation of the dynamical matrices computed on a 5 × 5 × 1 **q**-point mesh.

### Quasiparticles and Excitons.

Many-body calculations (34, 57, 58) were performed by using the Yambo code (59, 60). Quasiparticle corrections to the Kohn–Sham energies were evaluated using the $G^{0}W^{0}$ approximation for the self-energy, the dynamical dielectric screening being accounted for within the plasmon-pole approximation (61). To speed up the convergence of quasiparticle energies with respect to the number of empty bands in the sum over states occurring in the calculation of the polarizability and the self-energy, we have adopted the scheme proposed in ref. 62. Fifty empty bands were used to build the polarizability and to integrate the self-energy (*SI Appendix*, Fig. S10); the Brillouin zone was sampled by using a 27 × 27 × 3 **k**-point grid. Quasiparticle energies were converged by using 68- and 15-Ry kinetic energy cutoffs for the exchange and correlation parts of the self-energy (*SI Appendix*, Fig. S11), respectively. Excitation energies and dispersion of the lowest exciton with finite wavevector **q** were calculated by solving the Bethe–Salpeter equation (BSE) using the Yambo code where the finite-**q** BSE was implemented as described in refs. 63 and 64. The static screening in the direct term was calculated within the random phase approximation with the inclusion of local field effects; the Tamm–Dancoff approximation for the Bethe–Salpeter Hamiltonian was employed, after having verified that the correction introduced by coupling the resonant and the antiresonant part was negligible for **q** = 0. Converged excitation energies were obtained considering respectively three valence and five conduction bands in the Bethe–Salpeter matrix, the irreducible Brillouin zone being sampled with a 27 × 27 × 3 **k**-point grid (*SI Appendix*, Fig. S12).

### Computational Details of the Two-Band Model.

The effective-mass framework builds on the knowledge of conduction

$$\varepsilon_{b}\left(k\right)=\frac{G}{2}+\frac{\hbar^{2}}{2}\left[\frac{{\left(k_{∥}+\Gamma \Lambda \right)}^{2}}{m_{b∥}}+\frac{k_{⊥}^{2}}{m_{b⊥}}+\frac{k_{z}^{2}}{m_{bz}}\right]$$ [6]

and valence

$$\varepsilon_{a}\left(k\right)=-\frac{G}{2}-\frac{\hbar^{2}}{2}\left[\frac{k_{∥}^{2}}{m_{a∥}}+\frac{k_{⊥}^{2}}{m_{a⊥}}+\frac{k_{z}^{2}}{m_{az}}\right]$$ [7]

energy bands. Here G>0 (G<0) is the indirect bandgap (band overlap) for pressures below (above) the semiconductor–semimetal threshold—in the absence of excitonic effects—and the momentum components, $k_{∥}$, $k_{⊥}$, $k_{z}$, are projected along the principal axes of the effective mass tensor (65), the corresponding masses being $m_{i∥}$, $m_{i⊥}$, $m_{iz}$, with i=a,b. These axes are respectively parallel ($k_{∥}$) and perpendicular (in-plane ($k_{⊥}$) and out-of-plane ($k_{z}$)) to the $\vec{\Gamma \Lambda }$ direction, the axis origin being placed at the band edge. We emphasize that all parameters of the two-band model, for a given pressure, are fixed and obtained from first principles. In particular, the bandgap and the effective masses are extracted from GW bands, as illustrated in Fig. 2 *D*–*F*, and hence include the mean-field renormalization due to e-e interactions. The (modulus) of the screened e-h Coulomb attraction in momentum space,

$$W\left(q\right)=\frac{1}{\kappa_{r}}\frac{4\pi e^{2}}{\Omega }\frac{1}{q^{2}},$$ [8]

depends on the static dielectric constant, $\kappa_{r}$, which is obtained as the inverse of the first-principles dielectric tensor, $1/{\left[\epsilon^{-1}\left(q=0\right)\right]}_{G=G^{\prime }=0}$, in the long-wavelength, macroscopic limit, as illustrated in Fig. 3*B* (here Ω is the crystal volume and **G** the reciprocal lattice vector).

In the semimetal, the P-dependent values of G, $m_{i∥}$, $m_{i⊥}$, $m_{iz}$, and $\kappa_{r}$ are derived as linear extrapolations of first-principles data at P= 25 and 34 GPa, respectively. Since free e and h carriers effectively screen the interaction by adding a metal-like, intraband contribution to the polarizability, we modify the dressed Coulomb potential as

$$W\left(q\right)=\frac{1}{\left[\kappa_{r}+4\pi e^{2}D\left(\varepsilon_{\text{F}}\right)/q^{2}\right]}\frac{4\pi e^{2}}{\Omega }\frac{1}{q^{2}}.$$ [9]

Here the Thomas–Fermi term, proportional to the density of states, D(ε), evaluated at the Fermi energy, $\varepsilon_{\text{F}}$, removes the long-wavelength divergence of W. We obtain numerically D through the summation of Gaussian functions over a fine grid in **k** space, as well as $\varepsilon_{\text{F}}$ by imposing overall charge neutrality (we take into account the sixfold degeneracy of the conduction band).

### Two-Band Bethe–Salpeter Equation.

In the semiconductor, the exciton wave function is

$$\left|\text{exciton}\right\rangle =\underset{k}{\sum }\phi_{k}\;{\hat{b}}_{k}^{+}{â}_{k}\left|\Phi_{0}\right\rangle ,$$ [10]

where $\phi_{k}$ is the probability amplitude of a bound e-h pair in momentum space. The Bethe–Salpeter equation of motion for $\phi_{k}$ is

$$\left[\varepsilon_{b}\left(k\right)-\varepsilon_{a}\left(k\right)\right]\phi_{k}-\underset{k^{\prime }}{\sum }W\left(k-k^{\prime }\right)\phi_{k^{\prime }}\;=\varepsilon_{\text{exc}}\;\phi_{k},$$ [11]

where $\varepsilon_{\text{exc}}$ is the excitation energy of the exciton, whose negative value signals the instability. We solve this equation by numerical discretization in **k** space and assess convergence by refining the mesh as well as varying the momentum cutoff. Note that the singularity of Coulomb potential for $\left|q\right|\to 0$ is harmless, as we integrate W over a small parallelepiped, in a semianalytical, accurate manner. We have benchmarked the convergence of our calculations against known analytical or high-precision results, as shown for bulk Wannier excitons in *SI Appendix*, Fig. S13 and for anisotropic excitons with a well-defined azimuthal quantum number (66) in *SI Appendix*, Fig. S14.

In the semimetal ground state, a small area of **k** space around the origin is populated by electrons in band b and holes in band a. In addition, due to band anisotropy (67), nearby regions exist populated by either electrons or holes only, which prevents from exciting e-h pairs due to Pauli exclusion principle. Therefore, the Bethe–Salpeter equation of motion must be modified as (4)

$$\left[\varepsilon_{b}\left(k\right)-\varepsilon_{a}\left(k\right)\right]\phi_{k}-\underset{k^{\prime }}{\sum }W\left(k-k^{\prime }\right)\left[n_{a}\left(k^{\prime }\right)-n_{b}\left(k^{\prime }\right)\right]\phi_{k^{\prime }}\;=\varepsilon_{\text{exc}}\;\phi_{k},$$ [12]

where $n_{i}\left(k\right)$ is the occupancy factor of the ith band in the normal ground state, which takes either 0 or 1 as a value. The “counting” prefactor of W, $\left[n_{a}-n_{b}\right]$, removes scattering channels forbidden by Pauli blocking and is responsible of the plasmon-like features shown Fig. 5*E*. Note that in the semiconductor, $n_{a}\left(k\right)=1$ and $n_{b}\left(k\right)=0$, and hence one regains the standard form of Eq. **11**.

### Self-Consistent Theory of the Excitonic Insulator within the Two-Band Model.

The EI bands (circles in Fig. 4*A*) are $E_{bk}=\left[\varepsilon_{b}\left(k\right)+\varepsilon_{a}\left(k\right)\right]/2+E_{k}$ and $E_{ak}=\left[\varepsilon_{b}\left(k\right)+\varepsilon_{a}\left(k\right)\right]/2-E_{k}$, with $E_{k}$ being fixed by the solution of the gap Eq. **3** for $\Delta_{k}^{0}$ (through $\zeta_{k}^{0}$). Eq. **3** is solved self-consistently by numerical recursion, exploiting the exciton wave function $\phi_{k}$ as a seed (35). If the semimetal is the normal ground state, the gap equation maintains the form 3 of the main text, provided that 1) the summation over **k**${}^{\prime }$ is limited to those points whose occupancies are such that $\left[n_{a}\left(k^{\prime }\right)-n_{b}\left(k^{\prime }\right)\right]\neq 0$ to comply with Fermi statistics (67) and 2) the dressed Coulomb interaction W is renormalized by a vertex correction associated with the EI ground state (48), as the opening of the many-body gap significantly enhances the e-h attraction—by suppressing screening—with respect to the gapless normal phase. Therefore, following Kozlov and Maksimov (48), for small momentum transfer q the dressed interaction W appearing in Eq. **3** takes the self-consistent form

$$W\left(q\right)=\frac{1}{\left[1+\alpha /{\left(\Delta_{k_{\text{F}}}^{0}\right)}^{2}\right]}\frac{1}{\kappa_{r}}\frac{4\pi e^{2}}{\Omega }\frac{1}{q^{2}},$$ [13]

where the gap function at the Fermi surface, $\Delta_{k_{\text{F}}}^{0}$, which is determined recursively, removes the long-wavelength divergence as one approaches the EI–semimetal boundary. Here $\Delta_{k_{\text{F}}}^{0}$ is an average value defined as $\Delta_{k_{\text{F}}}^{0}={\left[\Delta_{k_{x\text{F}},0,0}^{0}\Delta_{0,k_{y\text{F}},0}^{0}\Delta_{0,0,k_{z\text{F}}}^{0}\right]}^{1/3}$, with $k_{x\text{F}}$ given implicitly by $\varepsilon_{\text{F}}=\varepsilon_{b}\left(k_{x\text{F}},0,0\right)$, and similarly for $k_{y\text{F}}$ and $k_{z\text{F}}$. The constant α, for given band overlap G<0, is $\alpha ={\left[\left|G_{0}\right|{\left(\varepsilon_{\text{F}}-G/2\right)}^{3/2}\right]}^{1/2}$, where $\left|G_{0}\right|=9.38$ meV is the maximum magnitude of the band overlap at which e-h pairing takes place. We neglect the modification of Eq. **13** for large momentum transfer, as it turns out to be irrelevant numerically. Whereas the vertex form 13 was originally proposed (48) for the case of spherically symmetric e and h pockets, we note that, at the semiconductor–semimetal threshold, the exciton responsible for the instability is essentially isotropic (*SI Appendix*, Fig. S5). At finite temperature, T, the gap equation takes the form

$$2E_{k}\;\zeta_{k}^{0}-\underset{k^{\prime }}{\sum }W\left(k-k^{\prime }\right)\;\zeta_{k^{\prime }}^{0}\left[f_{\text{F}}\left(E_{ak^{\prime }}-\varepsilon_{\text{F}}\right)-f_{\text{F}}\left(E_{bk^{\prime }}-\varepsilon_{\text{F}}\right)\right]\;=\;0,$$ [14]

where $f_{\text{F}}\left(x\right)=1/\left[1+\exp\left(\beta x\right)\right]$ is the Fermi distribution function, with $\beta =1/k_{\text{B}}T$ and $k_{\text{B}}$ being the Boltzmann constant, and we neglect the small renormalization of the chemical potential due to the presence of the exciton condensate.

### Multivalley Band Structure.

The calculation of the EI band structure relies on the theory by Monney et al. (46) to include valley degeneracy. This approach, based on Green functions, generalizes to multiple bands the original theory by Jérome et al. (3). For every **k** point, the EI band energies (solid lines in Fig. 4*A* and *SI Appendix*, Fig. S7) are found as the seven roots of the equation

$$z-\varepsilon_{a}\left(k\right)-\overset{6}{\underset{i=1}{\sum }}\frac{{\left|\Delta_{i}\left(k\right)\right|}^{2}}{z-\varepsilon_{ib}\left(k\right)}=0$$ [15]

(cf. equation 8 of ref. 46), after the magnitudes of the excitonic gap components, $\Delta_{i}\left(k\right)$, are obtained as follows. The gap function is defined as

$$\Delta_{i}\left(p\right)=\underset{k}{\sum }W\;\left(k\right)\;\zeta_{ik+p},$$ [16]

with $\zeta_{ik}$, apart from a phase factor, being the equal-time interband excitonic coherence $F_{i}^{†}\;\left(k,t,t\right)$ defined in equation 4 of ref. 46,

$$\zeta_{ik}=-iF_{i}^{†}\;\left(k,t+\delta ,t\right)=\frac{1}{2\pi i}\int_{-\infty }^{\infty }\;\text{d}\omega \;F_{i}^{†}\;\left(k,\omega \right)\;{\text{e}}^{-i\omega \delta },$$ [17]

and $\delta \to 0^{+}$ being a positive infinitesimal quantity. The integral 17 is evaluated through contour integration, the Fourier transform $F_{i}^{†}\;\left(k,\omega \right)$ being derived from the equations of motion of Green functions (46) as

$$\begin{array}{ccc} F_{i}^{†}\;\left(k,\omega \right)=-\Delta_{i}\left(k\right){\left[\omega -\varepsilon_{a}\left(k\right)-\underset{j\neq i}{\sum }\frac{{\left|\Delta_{j}\left(k\right)\right|}^{2}}{\omega -\varepsilon_{jb}\left(k\right)}\right]}^{-1}\times & & \\ {\left[\omega -\varepsilon_{ib}\left(k\right)-{\left|\Delta_{i}\left(k\right)\right|}^{2}{\left(\omega -\varepsilon_{a}\left(k\right)-\underset{j\neq i}{\sum }\frac{{\left|\Delta_{j}\left(k\right)\right|}^{2}}{\omega -\varepsilon_{jb}\left(k\right)}\right)}^{-1}\right]}^{-1}. & & \end{array}$$ [18]

Whereas this expression would generically lead to an intractable system of six coupled equations for the $\Delta_{i}$s, we exploit the high symmetry of the problem to simplify the form of $F_{i}^{†}\;\left(k,\omega \right)$ and recover a single gap equation. As discussed in the main text and *SI Appendix*, Fig. S6, the symmetrizing effect of e-h attraction makes $\Delta_{k}^{0}$ almost independent from the azimuthal angle $\varphi_{k}$, with **k** $\equiv \left(k,\varphi_{k},k_{z}\right)$ being expressed in cylindrical coordinates (k is the in-plane radial distance and $k_{z}$ the component along the z axis). Therefore, it is natural to assume that $\Delta_{i}$ has cylindrical symmetry, $\Delta_{i}\left(k\right)=\Delta \left(k,k_{z}\right){\text{e}}^{i\varphi_{i}}$. Since we are mainly interested in the region k≈0, we also neglect the azimuthal dependence of $\varepsilon_{ib}\left(k\right)$ in the denominator of $F_{i}^{†}$, obtaining

$$F_{i}^{†}\;\left(\omega \right)=-\frac{\Delta_{i}}{\left(\omega -\varepsilon_{a}-\frac{5{\left|\Delta \right|}^{2}}{\omega -\varepsilon_{ib}}\right)\left(\omega -\varepsilon_{ib}-\frac{{\left|\Delta \right|}^{2}}{\omega -\varepsilon_{a}-\frac{5{\left|\Delta \right|}^{2}}{\omega -\varepsilon_{ib}}}\right)},$$ [19]

where we omitted the dependence of terms on **k** in the notation. Eq. **19** is now easily integrated, giving a single self-consistent gap equation. This has the same form as Eq. **3** of the two-band model, provided that $\Delta_{k}^{0}$ is replaced with $\sqrt{6}\;\Delta_{i}\left(k\right)$.

### Ground-State Wave Function.

The contour integration of equal-time Green functions provides us with all interband coherences and band populations, i.e., $\left\langle \Psi_{\text{EI}}\right|{\hat{b}}_{jk}^{+}{\hat{b}}_{ik}\left|\Psi_{\text{EI}}\right\rangle =\Delta_{i}*\Delta_{j}/2E\left(E+\varepsilon_{b}/2-\varepsilon_{a}/2\right)$, $\left\langle \Psi_{\text{EI}}\right|{\hat{b}}_{ik}^{+}{\hat{b}}_{ik}\left|\Psi_{\text{EI}}\right\rangle ={\left(v^{0}\right)}^{2}/6$, $\left\langle \Psi_{\text{EI}}\right|{â}_{k}^{+}{â}_{k}\left|\Psi_{\text{EI}}\right\rangle ={\left(u^{0}\right)}^{2}$, where we omitted the dependence of right-hand-side terms on **k** to ease the notation, neglected the in-plane anisotropy of the valence band, $\varepsilon_{ib}=\varepsilon_{b}$, and put $E={\left\{{\left[\varepsilon_{b}-\varepsilon_{a}\right]}^{2}/4+|\Delta^{0}{|}^{2}\right\}}^{1/2}$. This allows us to write explicitly the ground-state wave function,

$$\left|\Psi_{\text{EI}}\right\rangle =\underset{k}{\prod }{\hat{\gamma }}_{k}^{+}\left|\text{vacuum}\right\rangle \;,$$ [20]

in terms of Bogoliubov–Valatin-like creation operators, ${\hat{\gamma }}^{+}$, which are defined as

$${\hat{\gamma }}_{k}^{+}=u_{k}^{0}\;{â}_{k}^{+}+\frac{v_{k}^{0}}{\sqrt{6}}\overset{6}{\underset{i=1}{\sum }}{\text{e}}^{-i\varphi_{i}}\;{\hat{b}}_{ik}^{+}.$$ [21]

As discussed in the main text, time reversal symmetry limits the number of independent condensate phases to three: $\varphi_{1}$, $\varphi_{3}$, and $\varphi_{5}$ (recall that $u_{k}^{0}=u_{-k}^{0}$ and $v_{k}^{0}=v_{-k}^{0}$ are real positive quantities) (*SI Appendix*, Fig. S6*C*).

### Inversion Symmetry Breaking.

The ground-state wave function allows us to understand the symmetry breaking associated with exciton condensation. The inversion operator, $\hat{I}$, acts differently on $b_{i}$ and a Bloch states, since the envelope function at Γ is odd: $\hat{I}{â}_{k}^{+}=-{â}_{-k}^{+}$, $\hat{I}{\hat{b}}_{1k}^{+}={\hat{b}}_{4-k}^{+}$, etc. Therefore, the inverted ground state, $\hat{I}\left|\Psi_{\text{EI}}\right\rangle$, is not proportional to the original one:

$$\begin{array}{ccc} & & \left\langle \Psi_{\text{EI}}\right|\hat{I}\left|\Psi_{\text{EI}}\right\rangle =\underset{k}{\prod }\{-{\left(u_{k}^{0}\right)}^{2}\\ & + & \frac{{\left(v_{k}^{0}\right)}^{2}}{3}\left[\cos\left(2\varphi_{1}\right)+\cos\left(2\varphi_{3}\right)+\cos\left(2\varphi_{5}\right)\right]\}. \end{array}$$ [22]

The magnitude of the expression enclosed in curly brackets is less than one (unless $\varphi_{1}=\varphi_{3}=\varphi_{5}=\pm \pi /2$, i.e., $\hat{I}\left|\Psi_{\text{EI}}\right\rangle =-\left|\Psi_{\text{EI}}\right\rangle$); hence, in the thermodynamic limit, the overlap between $\hat{I}\left|\Psi_{\text{EI}}\right\rangle$ and $\left|\Psi_{\text{EI}}\right\rangle$ tends to zero as the two states become orthogonal. Since the ground state has a lower symmetry than the Hamiltonian, inversion symmetry is broken.

### Charge Density Wave.

The form 5 of the purely electronic charge density wave, $\Delta \varrho =\Delta \varrho_{1,4}+\Delta \varrho_{3,6}+\Delta \varrho_{5,2}$, is derived in a straightforward manner by averaging the density operator, $\hat{\varrho }\left(r\right)=2\;{\hat{\psi }}^{†}\;\left(r\right)\;\hat{\psi }\left(r\right)$, over $\left|\Psi_{\text{EI}}\right\rangle$, with the Fermi field operator, $\hat{\psi }\left(r\right)$, being defined as

$$\hat{\psi }\left(r\right)=\underset{k}{\sum }{\text{e}}^{ik\cdot r}\left[\psi_{\Gamma }\;\left(r\right)\;{â}_{k}+\overset{6}{\underset{i=1}{\sum }}\exp\left(i{\vec{\Gamma \Lambda }}_{i}\cdot \text{r}\right)\psi_{\Lambda_{i}}\;\left(r\right)\;{\hat{b}}_{ik}\right].$$ [23]

Cross-terms proportional to $\psi {*}_{\Lambda_{i}}\psi_{\Lambda_{j}}$ average out to zero, once summed together, as the various $\psi_{\Lambda_{i}}$s are obtained one from the other by either rotation by ±2π/3 along the z axis or complex conjugation. Apart from the envelope functions, which have the lattice periodicity, Δϱ depends on **r** through a sum over three exponentials, whose imaginary arguments are respectively (times the prefactor i) ${\vec{\Gamma \Lambda }}_{1}\cdot r-\varphi_{1}$, ${\vec{\Gamma \Lambda }}_{3}\;\cdot \;r-\varphi_{3}$, and ${\vec{\Gamma \Lambda }}_{5}\;\cdot \;r-\varphi_{5}$, as illustrated in the main text.

We show below that, for any given two condensate phases, say $\varphi_{1}$ and $\varphi_{3}$, there exist a lattice vector $R_{\text{shift}}$ and a phase $\varphi_{5}=-\varphi_{1}-\varphi_{3}$ such that a rigid translation of the density wave by $R_{\text{shift}}$ provides the density wave corresponding to $\varphi_{1}=\varphi_{3}=\varphi_{5}=0$; i.e., ${\left[\Delta \varrho \left(r-R_{\text{shift}}\right)\right]}_{\varphi_{1},\varphi_{3},\varphi_{5}}={\left[\Delta \varrho \left(r\right)\right]}_{0,0,0}$.

Let us construct explicitly $R_{\text{shift}}$ as $R_{\text{shift}}=-R_{∥}-R_{⊥}$, where $R_{∥}=n_{∥}t_{2}$ and $R_{⊥}=n_{⊥}\left(2t_{1}+t_{2}\right)$ are respectively parallel and perpendicular to ${\vec{\Gamma \Lambda }}_{1}$ (*SI Appendix*, Fig. S6*C*); $n_{∥}$ and $n_{⊥}$ are integers to be determined; and $t_{1}$, $t_{2}$ are the primitive vectors that generate the hexagonal lattice in Mattheiss’s (47) coordinate frame. Since ${\vec{\Gamma \Lambda }}_{1}$ is generically not commensurable with the reciprocal lattice vectors, there exists an integer $n_{∥}$ such that ${\vec{\Gamma \Lambda }}_{1}\cdot R_{∥}=\varphi_{1}$ with arbitrary accuracy (4), modulus an integer multiple of 2π. Similarly, we may fix $n_{⊥}$ such that ${\vec{\Gamma \Lambda }}_{3}\cdot R_{⊥}=-{\vec{\Gamma \Lambda }}_{5}\cdot R_{⊥}=\varphi_{3}-{\vec{\Gamma \Lambda }}_{3}\cdot R_{∥}$. Finally, we take $\varphi_{5}=-\varphi_{3}+2{\vec{\Gamma \Lambda }}_{3}\cdot R_{∥}=-\varphi_{3}-\varphi_{1}$. One may verify, by direct substitution into the expression $\Delta \varrho =\Delta \varrho_{1,4}+\Delta \varrho_{3,6}+\Delta \varrho_{5,2}$, that ${\left[\Delta \varrho \left(r-R_{\text{shift}}\right)\right]}_{\varphi_{1},\varphi_{3},-\varphi_{1}-\varphi_{3}}={\left[\Delta \varrho \left(r\right)\right]}_{0,0,0}$, Q.E.D.

This theorem implies that the set of charge density waves ${\left[\Delta \varrho \left(r\right)\right]}_{0,0,\varphi_{5}}$ labeled by the continuous parameter $\varphi_{5}$ spans all possible modulations of the electronic charge density of the EI, each realization having in turn a huge translational degeneracy, which is parameterized by the two continuous variables $\varphi_{1}$ and $\varphi_{3}$.

### Antiferroelectric Order.

The electronic charge density wave of the EI ground state (Eq. **5**) induces an out-of-plane electric dipole, $P_{z}\left(R_{i}\right)$, in the ith cell of the pristine 2H phase located at $R_{i}$, with i=1,…,N (N is the total number of cells). This is illustrated in Fig. 4*B*, where the dipoles $P_{z}\left(R_{i}\right)$ are depicted as red arrows. The local dipole $P_{z}\left(R_{i}\right)$ is given by the coherent superposition of three density waves, whose characteristic wave vectors are $q_{i}={\vec{\Gamma \Lambda }}_{i}$, with i=1,3,5,

$$P_{z}\left(R_{i}\right)=\frac{P_{z0}}{\Omega }\underset{k}{\sum }\frac{4}{\sqrt{6}}u_{k}^{0}v_{k}^{0}\;\underset{j=1,3,5}{\sum }\cos\left(q_{j}\cdot R_{i}\right).$$ [24]

The maximum value, $P_{z}\left(0\right)$, is shown in Fig. 4*D*. Here $P_{z}\left(R_{i}\right)$ is evaluated within the envelope function approximation, the factor $P_{z0}$ being derived from first principles through the overlap charge density of the periodic part of conduction and valence Bloch states at Γ and Λ, respectively, which is shown in Fig. 4*C*. The latter is numerically integrated over the pristine unit cell volume, $\Omega_{\text{cell}}$:

$$P_{z0}=\underset{\sigma }{\sum }e\;\int_{\Omega_{\text{cell}}}\;dr\;z\left[\psi {*}_{\Gamma \sigma }\left(r\right)\;\psi_{\Lambda \sigma }\left(r\right)\;+\;\text{c.c.}\right],$$ [25]

the frame origin being placed at the inversion center—the midpoint between the two Mo atoms of the 2H cell. As the charge displacement that gives rise to the dipole is essentially localized on Mo atoms (Fig. 4*C*), we expect $\left|P_{z0}\right|$ to be well defined. We obtain $P_{z0}/e=$ 15.1 Bohr at P=34 GPa.

## Supplementary Material

Supplementary File

## Data Availability

Many-body perturbation theory calculations were performed by means of the codes Yambo (www.yambo-code.org/) and Quantum ESPRESSO (www.quantum-espresso.org), which are both open source software. Results for the two-band model were obtained through a custom Fortran code that is available at Zenodo, doi.org/10.5281/zenodo.4455373. The data that support the findings of this study (crystal structures) are available at Zenodo, doi.org/10.5281/zenodo.4455373 (68).
