# Supplementary material for: Evidence of ideal excitonic insulator in bulk MoS2 under pressure
Source: Proc Natl Acad Sci U S A. 2021 Mar 23;118(13):e2010110118. doi: 10.1073/pnas.2010110118 (PMC8020749; doi:10.1073/pnas.2010110118)
Supplement: Supplementary File [file pnas.2010110118.sapp.pdf]

PNAS
www.pnas.org

Supplementary Information for

Evidence of ideal excitonic insulator in bulk MoS_2 under pressure

S. Samaneh Ataei, Daniele Varsano, Elisa Molinari and Massimo Rontani

Massimo Rontani.
E-mail: massimo.rontani@nano.cnr.it

This PDF file includes:

Figs. S1 to S14
SI References

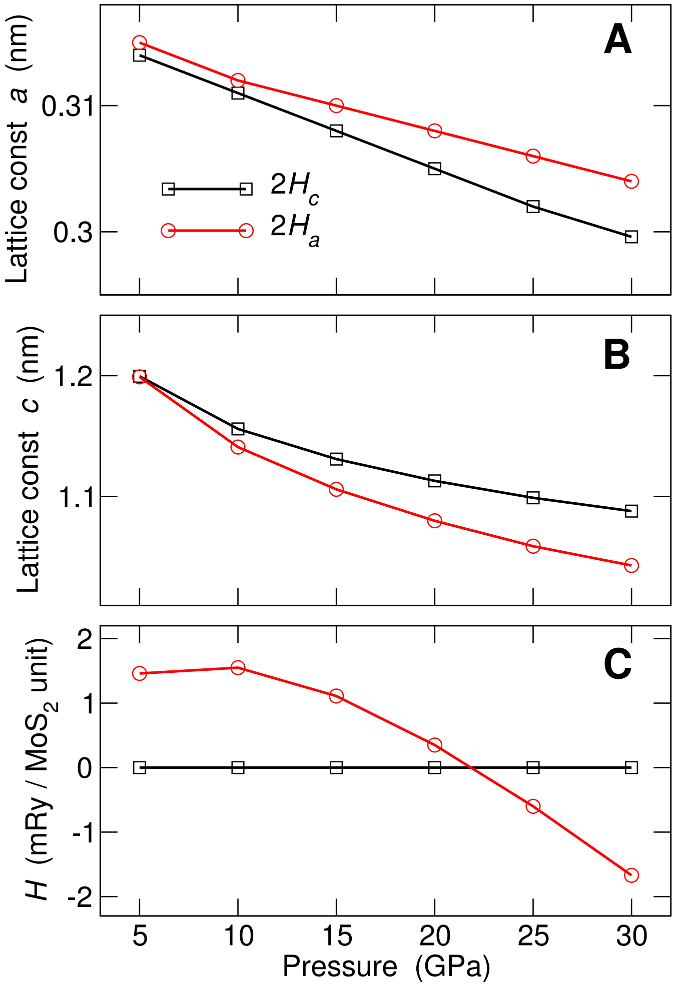

Fig. S1. Stability of $2H_c$ and $2H_a$ phases of MoS_2 vs pressure. (**A** and **B**) Structural parameters a (panel A) and c (panel B) of $2H_c$ (squares) and $2H_a$ (circles) phases of MoS_2 versus pressure, as obtained from first principles. The sketches of the corresponding crystal unit cells are shown in Fig. 1B and Fig. S2, respectively. (**C**) Relative enthalpy, H, of $2H_c$ (squares) and $2H_a$ (circles) phases vs pressure. Lines are guides to the eye. Computed lattice constants compare well with those reported in Fig. 2 of Ref. (1), whereas the transition from the $2H_c$ to the $2H_a$ phase is expected at a slightly larger pressure (22 vs 20 GPa).

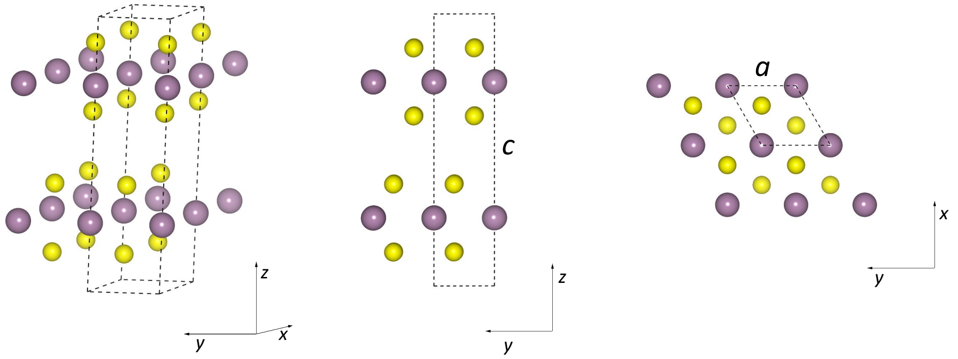

Fig. S2. Crystal structure of the $2H_a$ phase of MoS_2. Model of the $2H_a$ crystal structure from different views. The violet (yellow) colour labels Mo (S) atoms. The dashed frame appearing in side and top views is the unitary cell of the layered structure, with a and c being the in- and out-of-plane lattice constants, respectively.

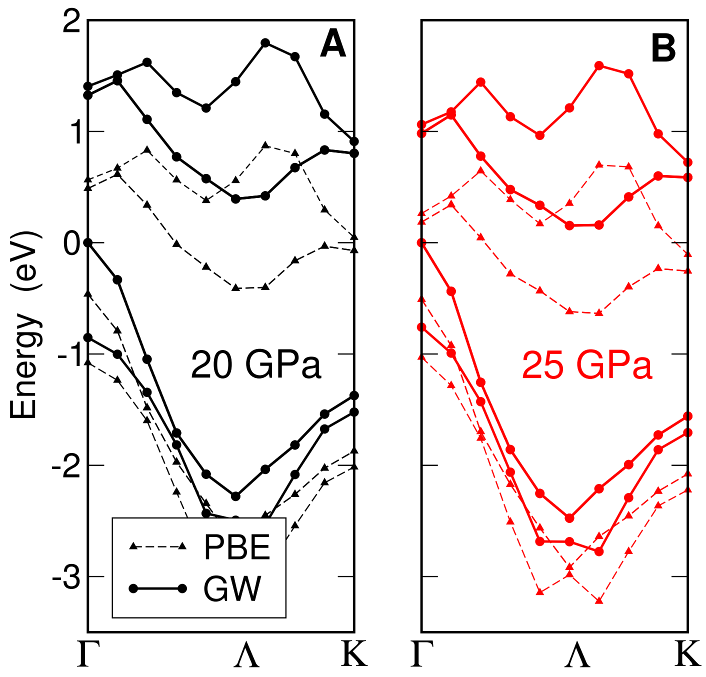

Fig. S3. Band structure of the $2H_a$ phase of MoS_2 vs pressure. (**A** and **B**) Band structure of the $2H_a$ phase along the $\Gamma - \Lambda -$ K cut of the Brillouin zone at pressure $P =$ 20 (panel A) and 25 GPa (panel B). Band energies obtained from first principles including the quasiparticle GW corrections beyond DFT (circles) are compared to bare DFT data (triangles, PBE functional). Lines are guides to the eye. Note that the lowest conduction and highest valence GW bands at 25 GPa are almost identical to those of the $2H_c$ phase (Fig. 2B). The extrapolated value of pressure at which the GW indirect gap closes is 27.6 GPa.

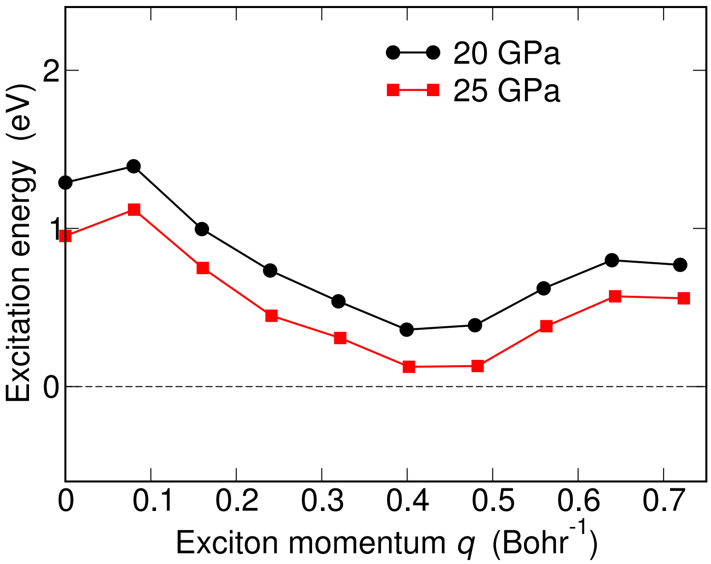

Fig. S4. Exciton dispersion for the $2H_a$ phase of MoS_2. Excitation energy of the lowest exciton vs center-of-mass momentum $\mathbf{q}$ along the ΓK direction for the $2H_a$ phase of MoS_2. Data are obtained from first principles for $P = 20$ (dots) and 25 GPa (squares). Note that the K point position expressed in units of Bohr^{-1} shifts with P. Lines are guides to the eye. The mimimum excitation energy extrapolated at the semimetal threshold (27.6 GPa, see Fig. S3) is -27 meV, the same value obtained for the $2H_c$ phase discussed in the main text, which shows that the excitonic instability does not depend on the $2H_c - 2H_a$ structural transition.

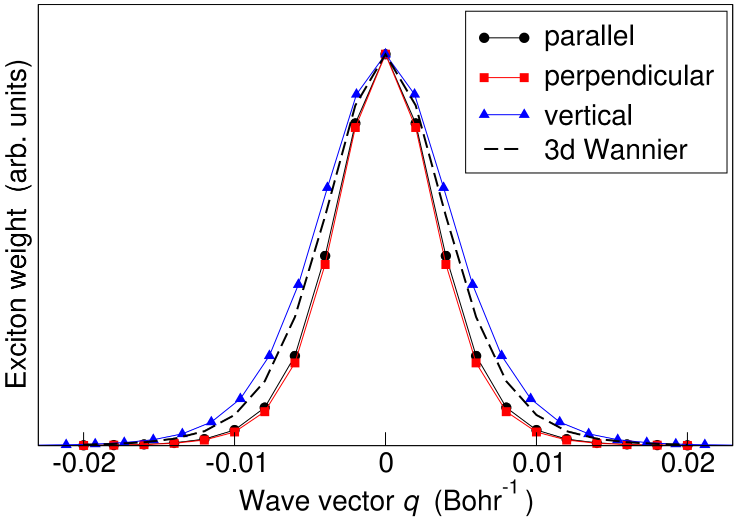

Fig. S5. Exciton probability weight along the principal axes of the effective mass tensor within the two-band model. Wave function square modulus of the exciton driving the instability in the e-h center-of-mass frame, obtained at a pressure of 34 GPa for the $2H_c$ phase from the two-band Bethe-Salpeter equation. Data, which are evaluated in reciprocal space, are probabilities to excite e-h pairs of wave vector $\mathbf{q}$, made of a hole with momentum $-\mathbf{q}$ and an electron with momentum $\mathbf{q}+\vec{\Gamma\Lambda}$. The probability map is shown along those cuts of the Brillouin zone parallel to the principal axes of the effective-mass tensor, i.e., parallel (circles) and perpendicular [in- (squares) and out-of-plane (triangles)] to the $\vec{\Gamma\Lambda}$ direction, respectively. The weight distribution, which is almost circularly symmetric in the plane, compares with that of the three-dimensional, fully isotropic Wannier exciton (dashed line). The Bohr radius of the latter is 50 Å and the mass, m_{Wannier}, is the algebraic mean of the masses along the three principal axes, $m_{\text{Wannier}} = (m_{\|}\, m_{\perp}\, m_z)^{1/3}$, with $m_{\|} = (1/m_{a\|} + 1/m_{b\|})^{-1}$ being the exciton mass along the $\vec{\Gamma\Lambda}$ direction, and similarly for the other axes.

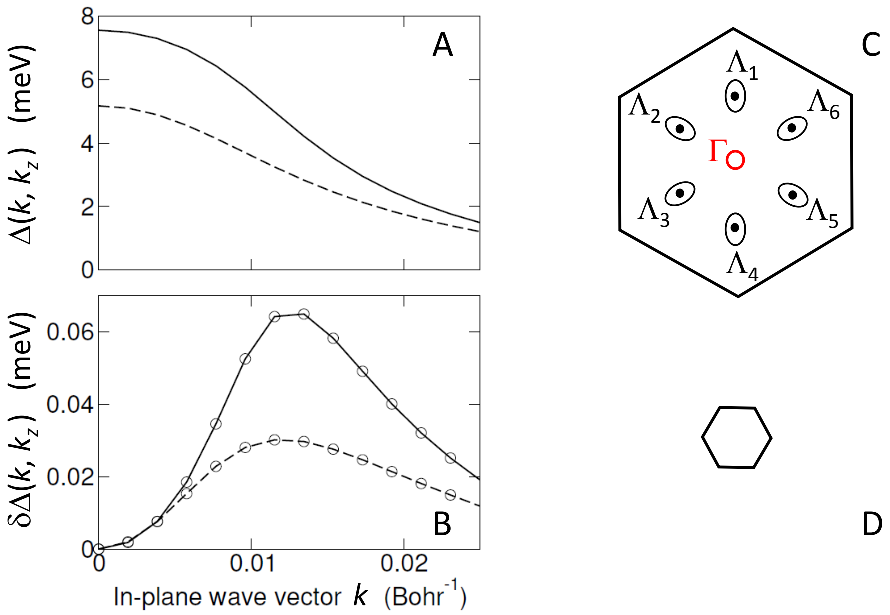

Fig. S6. Excitonic gap function. The excitonic gap function, $\Delta^0_{\mathbf{k}}$, slightly deviates from perfect cylindrical symmetry and hence may be interpolated in cylindrical coordinates as: $\Delta^0_{(k,\varphi_{\mathbf{k}},k_z)} = \Delta(k,k_z) + \delta\Delta(k,k_z)\,\cos(2\varphi_{\mathbf{k}})$, with k being the in-plane radial distance, $\varphi_{\mathbf{k}}$ the azimuthal angle, and k_z the component along the z axis. The first addendum, $\Delta(k,k_z)$, is the average radial profile, whereas the second one, $\delta\Delta(k,k_z)$, is the in-plane azimuthal modulation. The angle $\varphi_{\mathbf{k}} = 0$ corresponds to the in-plane direction parallel to the $\vec{\Gamma\Lambda}$ vector. (**A**) Average radial profile of the excitonic gap function, $\Delta(k,k_z)$, vs in-plane radial distance k for $k_z = 0$ (solid curve) and $k_z = 1.4 \cdot 10^{-2}$ Bohr^{-1} (dashed curve), at $P = 34$ GPa. (**B**) Azimuthal modulation of the excitonic gap function, $\delta\Delta(k,k_z)$, vs k for $k_z = 0$ (solid curve) and $k_z = 1.4 \cdot 10^{-2}$ Bohr^{-1} (dashed curve), at $P = 34$ GPa. Lines are guides to the eye. (**C**) Sketch of the Brillouin zone of the normal phase in the plane $k_z = 0$. The ellipses drawn in black (red) colour represent electron (hole) Fermi pockets in the semimetal. The ellipse eccentricity at valley bottoms Λ_i is purposely exaggerated for the sake of clarity ($i = 1, \ldots, 6$). (**D**) Sketch of the reconstructed Brillouin zone of the excitonic insulator in the plane $k_z = 0$. The coordinate frame has the same orientation and length scale in both panels C and D.

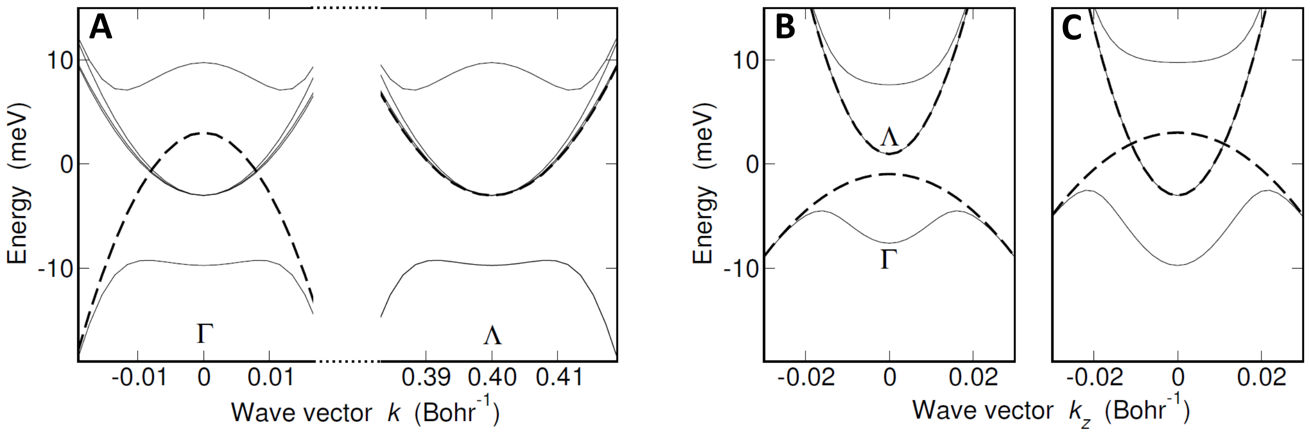

Fig. S7. Band structure of the excitonic insulator along different high-symmetry directions and for different pressures. (**A**) Band structure of the excitonic insulator (solid curves) and pristine semimetal (dashed curves) along one of the six equivalent $\Gamma - \Lambda$ directions in the $k_z = 0$ plane of the Brillouin zone at $P =$ 34.19 GPa. The original conduction bands are folded from Λ valleys to Γ, and renormalized together with the valence band. The new band structure at Γ is replicated at Λ, since both Γ and Λ points belong to the EI reciprocal lattice. Apart from spin degeneracy, EI renormalized bands exhibit an additional orbital degeneracy reminescent of the pristine multivalley structure: bands (solid curves) from top to bottom are respectively one-, three-, one-, one-, and one-fold degenerate, respectively. (**B-C**) Band structure of the excitonic insulator (solid curves) and normal phase (dashed curves) along the $\Gamma -$ A direction parallel to the k_z axis of the Brillouin zone at $P =$ 34.0 GPa (panel B) and 34.19 GPa (panel C), respectively. The normal phase of panel B (C) is the semiconductor (semimetal). The EI conduction band (solid curve) that overlaps with the pristine band (dashed curve) is five-fold degenerate. The pristine band has been rigidly translated by the wave vector $-\vec{\Gamma\Lambda}$. In both cases B and C the actual EI gap is indirect, as the valence band top is slightly displaced from Γ along the k_z axis. In panel C the excitonic gap closes whereas the amount of condensed excitons remains finite.

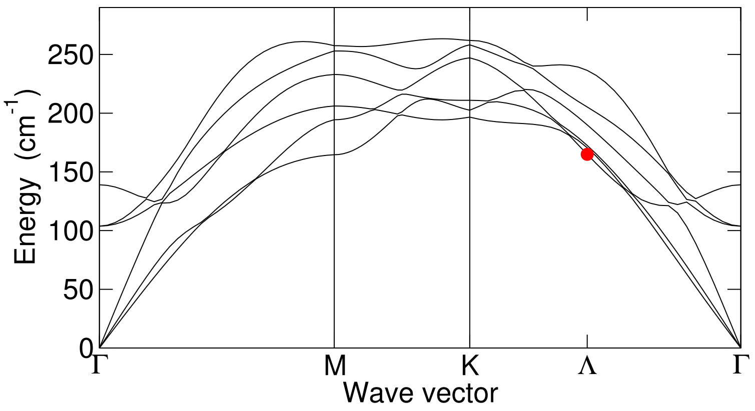

Fig. S8. **Phonon dispersion for the $2H_a$ phase of MoS_2 at a pressure of 34 GPa.** Dispersion of the lowest-energy phonon modes for $P = 34$ GPa computed from first principles for the $2H_a$ phase of MoS_2. The red dot points to the lowest optical mode that is folded from Λ into Γ through the excitonic insulator phase transition. Its frequency is almost identical to the corresponding value for the $2H_c$ phase (166 vs 164 cm^{-1}).

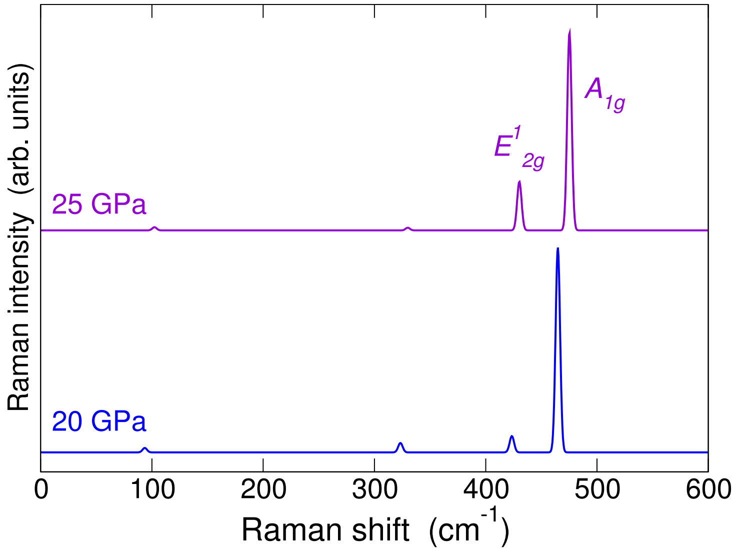

Fig. S9. Raman spectrum for the $2H_a$ normal phase of MoS_2 at pressures of 20 and 25 GPa. Raman spectrum of the normal phase from first principles, for pressures $P =$ 20, 25 GPa, respectively from bottom to top. The peaks are arbitrarily broadened using Gaussians with a standard deviation of 2 cm^{-1}. The bright peak at lower (higher) frequency has E^1_{2g} (A_{1g}) symmetry. The plot should be compared with Fig. 4(b) of Ref. (2).

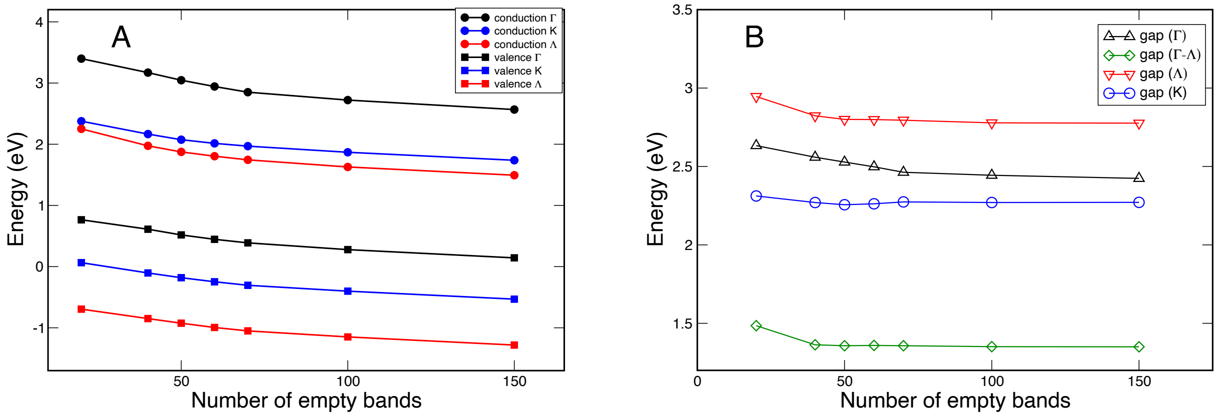

Fig. S10. Convergence of GW quasiparticle energies and gaps as a function of the number of empty bands used in the sum over states. (**A**) Valence and conduction quasiparticle energies at Γ, K, and Λ points. (**B**) Value of direct gaps at Γ, K, Λ, and indirect gap, $\Gamma\Lambda$, as a function of the number of empty bands used in the sum over states. The calculations were done at $P = 0$ GPa with 10 $\times$ 10 $\times$ 3 $\mathbf{k}$-point grid sampling and a 15 Ry energy cutoff for the dielectric matrix. In all calculations the technique to accelerate the convergence with respect to empty bands proposed by Bruneval and Gonze (3) was adopted. The usage of the acceleration technique allows us to obtain converged quasiparticle gaps already using $\sim$ 50 bands. Lines are guides to the eye.

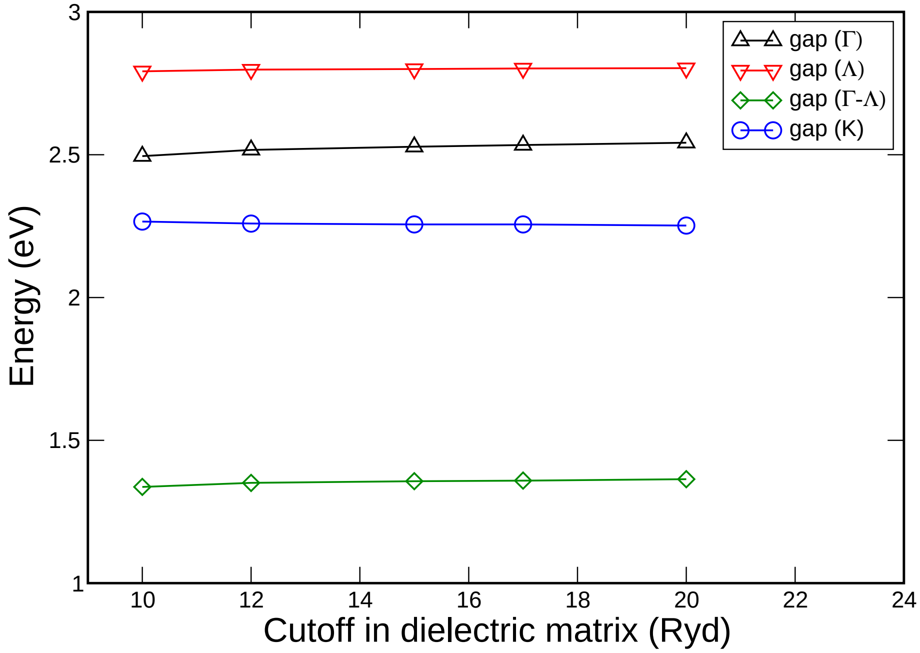

Fig. S11. Convergence of GW quasiparticle gaps as a function of the energy cutoff (matrix dimension) of the dielectric matrix. The calculations were done at $P = 0$ GPa with $10\times10\times3$ **k**-point grid sampling and the summation over 50 empty bands using the acceleration technique of Ref. (3). The convergence is achieved around 15 Ry. Lines are guides to the eye.

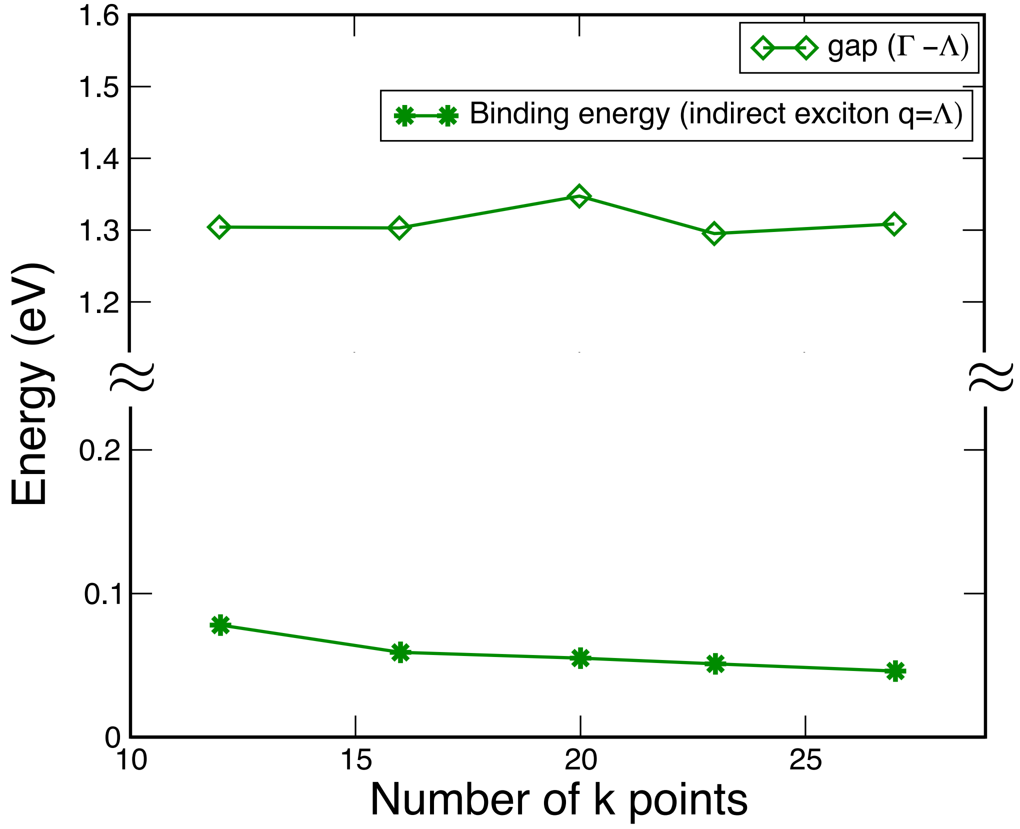

Fig. S12. Convergence of quasiparticle indirect gap (diamonds) and BSE results (stars) with respect the number of k points. The abscissa points to the number of **k** points along the x (y) direction. The convergence of the binding energy is already achieved for a $27 \times 27 \times 3$ **k**-point grid, including 3 valence and 5 conduction bands in the construction of the Bethe-Salpeter kernel. Lines are guides to the eye.

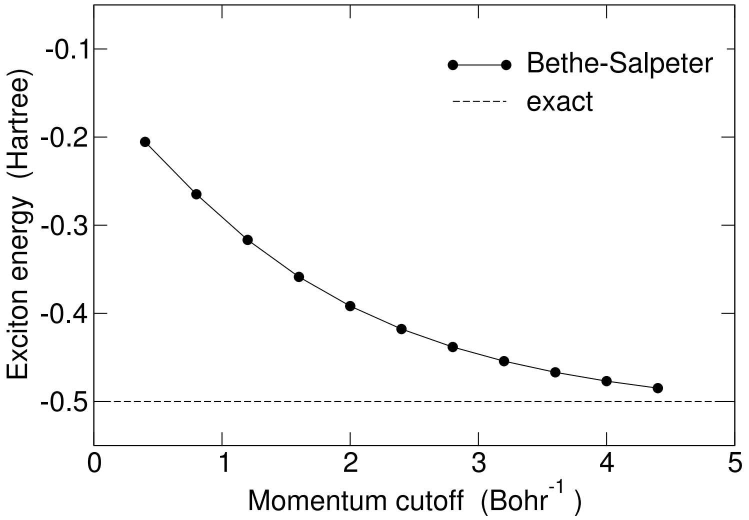

Fig. S13. Convergence of two-band Bethe-Salpeter calculation vs momentum cutoff. Energy of the bulk isotropic Wannier exciton versus momentum cutoff, as obtained from the two-band Bethe-Salpeter equation (circles). The eigenvalue problem is solved by diagonalizing a matrix defined over a cubic manifold in momentum space, as we use cartesian coordinates to fully take into account mass anisotropy. The momentum cutoff is the cube edge. The energy converges to its exact value (dashed line) for a sufficiently large momentum cutoff. Here $\kappa_r = 1$ and lines are guides to the eye.

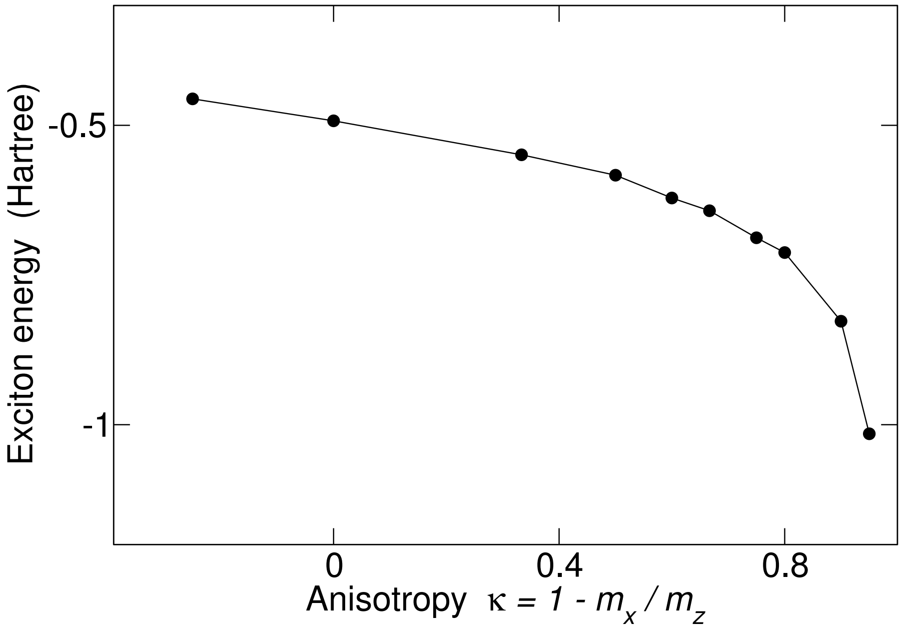

Fig. S14. Exciton energy vs anisotropy from the two-band Bethe-Salpeter calculation. Energy of the bulk Wannier exciton versus degree of out-of-plane mass anisotropy, κ, as obtained from the two-band Bethe-Salpeter equation (circles). The mass anisotropy, $\kappa = 1 - m_x/m_z$, is defined through the ratio of the in- (m_x) to out-of-plane (m_z) mass, the exciton wave function being circularly symmetric in the plane. Analytically known limits include the isotropic bulk ($m_x = m_z$, $\kappa = 0$) and two-dimensional ($m_z = \infty$, $\kappa = 1$) cases, having energies of $-1/2$ and -2 Rydberg, respectively (here $\kappa_r = 1$). Data match the accurate results reported in Fig. 2 of Ref. (4). Lines are guides to the eye.

References

1. L Hromadová, R Martoňák, E Tosatti, Structure change, layer sliding, and metallization in high-pressure mos$_2$. *Phys. Rev. B* **87**, 144105 (2013).
2. ZY Cao, JW Hu, AF Goncharov, XJ Chen, Nontrivial metallic state of MoS$_2$. *Phys. Rev. B* **97**, 214519 (2018).
3. F Bruneval, X Gonze, Accurate GW self-energies in a plane-wave basis using only a few empty states: Towards large systems. *Phys. Rev. B* **78**, 085125 (2008).
4. TG Pedersen, S Latini, KS Thygesen, H Mera, BK Nikolić, Exciton ionization in multilayer transition-metal dichalcogenides. *New J. Phys.* **18**, 073043 (2016).
